# Label-free multiphoton microscopy and machine learning for recognition of hepatocellular carcinoma

**DOI:** 10.1038/s41598-026-43831-y

**Published:** 2026-03-10

**Authors:** Roberta Galli, Sandra Korn, Daniela Aust, Gustavo B. Baretton, Jürgen Weitz, Edmund Koch, Carina Riediger

**Affiliations:** 1 Department of Medical Physics and Biomedical Engineering, Faculty of Medicine Carl Gustav Carus, Technische Universität Dresden, Fetscherstr. 74, 01307 Dresden, Germany; 2 Department of Visceral, Thoracic and Vascular Surgery, University Hospital Carl Gustav Carus, Technische Universität Dresden, Dresden, Germany; 3 Institute of Pathology, Medical Faculty, University Hospital Carl Gustav Carus, Technische Universität Dresden, Dresden, Germany; 4 National Center for Tumor Diseases (NCT/UCC), German Cancer Research Center (DKFZ), Dresden, Germany; 5 Clinical Sensoring and Monitoring, Department of Anesthesiology and Intensive Care Medicine, Faculty of Medicine Carl Gustav Carus, Technische Universität Dresden, Dresden, Germany

**Keywords:** Oncological surgery, Autofluorescence, Coherent anti-stokes Raman scattering, Second harmonic generation, Texture analysis, Cancer, Gastrointestinal cancer, Machine learning, Multiphoton microscopy, Fluorescence imaging, Molecular imaging

## Abstract

Complete tumor resection is crucial in oncological liver surgery, and the evaluation of intraoperative resection margins is essential to prove R0 resection. This can be challenging for hepatocellular carcinoma (HCC) due to the heterogeneity of both the tumor and background liver tissue. Label-free multiphoton microscopy (MPM) enables tissue analysis based on endogenous optical signals, and has the potential for intraoperative real-time assessment of resection planes. Matched samples of human HCC and background liver tissue from 76 patients were imaged using a multimodal approach, including coherent anti-Stokes Raman scattering, two-photon autofluorescence, and second harmonic generation. The morphological information contained in each channel was reduced to 17 texture parameters that were used for classification. A neural network model was trained on approximately 25,000 images (35 patients) and used to classify a test set of approximately 27,000 images (38 patients) as well as create maps showing the tumor border (3 patients). Label-free MPM revealed HCC growth patterns as well as steatotic and desmoplastic features. Accurate tumor recognition was achieved on low-lateral-resolution MPM images, mimicking the use of endoscopes. The model achieved a test set correct rate of 97.3% (98.2% for liver and 96.5% for tumor). Analysis of the contribution of the different nonlinear signals to the classification showed that autofluorescence plays a key role in discriminating between neoplastic and non-neoplastic tissue. In conclusion, label-free intraoperative optical histopathology of HCC has the potential to improve tumor resection margins. By implementation in endoscopes, MPM may enable on-site tissue analysis for optimization of tumor identification or characterization of liver tissue.

**Supplementary Information:**

The online version contains supplementary material available at 10.1038/s41598-026-43831-y.

## Introduction

Hepatocellular carcinoma (HCC) is one of the leading cancers worldwide and shows a high rate of cancer-related deaths^1^. HCC is often associated with underlying liver diseases with corresponding damage to the liver parenchyma. The main risk factors of HCC are viral hepatitis and liver cirrhosis. In recent years, non-alcoholic steatohepatitis has been identified as an independent risk factor for HCC, with increasing incidence^2^.

The basic principle of oncological liver surgery is complete tumor resection with tumor-free resection margins, which is essential for the patient’s oncological prognosis^3,4^. Because of the frequent association between HCC and damaged or altered liver parenchyma, evaluation of resection margins can be demanding. Especially in the setting of intraoperative fresh-frozen sections, differentiation between low-grade (G1) HCC and liver cirrhosis and steatosis is often not possible^5–7^. Moreover, intraoperative histopathological analyses are time-consuming, leading to prolonged operation times of up to 40 min. Consequently, new techniques for intraoperative real-time evaluation of resection margins are urgently required.

Multiphoton microscopy (MPM) is a powerful technique that allows the examination of tissue structures at a subcellular resolution by utilizing optical signals generated from endogenous tissue components upon irradiation with short-pulsed laser beams. In the last decade, this technique has gained increasing attention owing to its potential for rapid pathological assessment without tissue preparation. Notably, multimodal MPM has emerged as a significant advancement, enabling the simultaneous acquisition of various nonlinear optical signals from endogenous molecules, and has proven effective in the delineation of tumors^8,9^.

Typically, label-free MPM of the liver is based on imaging using at least two signals from coherent anti-Stokes Raman scattering (CARS), two-photon excited fluorescence (TPEF), and second harmonic generation (SHG) to visualize different tissue constituents. CARS allows imaging of lipid-rich tissue structures, such as cell membranes and lipid droplets, by displaying the distribution of C-H molecular groups. SHG enables the visualization of fibrillar collagen, for example in fibrotic tissue and blood vessels, while TPEF arises from other endogenous intra- and extra-cellular fluorophores such as elastin, NAD(P)H, FAD, and lipofuscin^10^.

Preclinical studies have utilized TPEF and SHG to diagnose liver cancer and differentiate between benign and malignant lesions, offering insights into tissue architecture and subcellular morphology^11^. Similar findings in rat models demonstrated the ability of TPEF and SHG for diagnosing liver cancer and lung metastases^12^. Leveraging deep learning-based automated classification of TPEF and SHG images achieved an accuracy above 90% in recognizing and grading HCC^13^.

Liver fibrosis was addressed extensively by visualizing collagen fibers using SHG microscopy, often supported by TPEF to recover cellular structures^14–19^. This approach was compared with established fibrosis scores and resulted successful for quantitative staging of liver fibrosis^20^. On the other side, steatosis was indirectly addressed by relying on the absence of nonlinear signals (e.g., TPEF) within the voids left by loss of lipid droplets after sectioning^21,22^. Multimodal imaging with CARS has been applied to direct analyze liver steatosis and fibrosis, enabling visualization of hepatic fats, collagen fibrils, and hepatocyte morphologies^23–26^. Furthermore, it has been demonstrated that the assessment of liver lipid levels based on the intensity of CARS signals correlates well with measurements of triglyceride levels obtained through a conventional biochemical assay^27^.

Automated image analysis, encompassing morphometric and texture analysis applied to label-free multiphoton images obtained by CARS, TPEF, and SHG, has emerged as an objective tool for tumor recognition, augmenting visual assessment by trained pathologists^28,29^. In the liver, it was shown that an approach based on label-free MPM integrating CARS, TPEF and SHG followed by texture feature extraction and linear discriminant analysis is able to recognize early septic injury in a murine model. In particular, CARS and TPEF demonstrated exceptional discriminative power^30^. In oncological applications, we recently showed that the combination of CARS, TPEF and SHG has the potential to increase the precision of resection margins in hepatic surgery of colorectal metastases. The morphological information with biochemical specificity provided by MPM allowed discrimination of the normal liver from metastases and discerning the tumor borders on unfixed tissue cryosections and formalin-fixed bulk tissue. Texture analysis followed by linear discriminant analysis attained a classification correct rate of 95% on over forty thousand MPM images of 106 patients^31^.

For in situ intraoperative applications, both photodamage and the availability of suitable endoscopic devices are of practical importance. The use of near-infrared laser excitation is recognized as an effective approach for mitigating photochemical alterations during MPM. In vivo experiments in animal models attested the lack of tissue damage under normal imaging settings, while any photochemical changes that might occur during MPM imaging can be readily identified across diverse tissue types, including the liver^32^. The feasibility of endoscopic label-free multimodal multiphoton microscopy has been demonstrated using both rigid endoscopes^33,34^ and optical fibers^35,36^. Commercial endoscopic systems for multiphoton microscopy are not yet ready for commercialization, and future developments should exploit the potential for further miniaturization.

This study aimed to investigate label-free multiphoton imaging techniques (CARS, TPEF, and SHG) for a comprehensive assessment of HCC, including recognition of distinct HCC subtypes based on their growth patterns and tissue microarchitecture. Furthermore, the research aimed at replicating the real-time functionality of intraoperative endoscopic devices by exploiting the rapid acquisition of low-resolution images using a laser-scanning microscope, thus emulating the lateral resolution of rigid CARS endoscopes that typically lies by approximately 1 μm^33,34^. In this perspective, the present study sought to bridge the gap between traditional ex vivo histopathology and in vivo imaging for future integration into surgical workflows. To enhance the power of the technique for supporting resection plane optimization, this study employed a neural network-based classification system for accurate tumor recognition. Another important aspect of the research involved investigating the contribution of each specific nonlinear optical signals to the accurate classification of HCC subtypes with the aim of providing a deeper understanding of the underlying tissue characteristics that drive successful tissue classification.

## Materials and methods

### Ethics statement and patient consent

The study was conducted in accordance with the Declaration of Helsinki and Istanbul, and approval was obtained from the local ethics committee of Technische Universität Dresden (BO-EK-26012020). All tissue samples were supplied by the Tumor and Normal Tissue Bank of the UCC Dresden and used in accordance with the rules of the tissue bank. Written informed consent was obtained from all patients. The study was registered at the German Clinical Trials Register (Trial ID: DRKS 00022261).

### Patients, database, and tissue samples

One hundred patients who underwent liver resection for HCC at the Department of Visceral, Thoracic, and Vascular Surgery at the University Hospital of Dresden between 2013 and 2018 were randomly selected from a prospective clinical database (containing 155 parameters per patient). Cryoconserved tissue samples of the selected patients were obtained from the tissue bank of the University Cancer Center (UCC) Dresden. Samples of matched liver and tumor tissue were available from 76 patients. Patient clinical information was retrieved from a clinical database. The majority of patients were males (63/76), the median age was 70 years (range: 20–85 years), and the median BMI was 28.4 kg/m^2^ (range: 18.4–44.2). More than half of the patients had diabetes mellitus (*n* = 46/76). Almost all patients (*n* = 71/76) had pathological liver changes to different extents (steatosis, steatohepatitis, fibrosis), and cirrhosis was diagnosed in approximately half of these patients (*n* = 38/71). Only one patient had a history of hepatitis B. Patient characteristics are reported in Supplementary Tables S1 – S3. The degree of tumor differentiation was retrieved in the frame of the routine histopathological diagnostics based on the Edmonson and Steiner grading scale (surgery before 2019) or on the WHO classification of liver tumors released in 2019^37^. Grade G1 corresponds to well-differentiated tumors, G2 to moderately differentiated tumors, and G3 to poorly differentiated tumors. The number of G1 samples was 8, of G2 was 49 and of G3 was 19.

Cryosections of unfixed frozen tissue with a thickness of 10 μm were prepared on glass slides and stored at -80 °C until microscopy imaging.

### Label-free multiphoton imaging

A laser scanning microscope (Axio Examiner Z.1 with scanning module LSM 7, Carl Zeiss AG, Jena, Germany) coupled with two picosecond lasers at 781 nm and 1005 nm (FemtoFiber pro NIR and FemtoFiber pro TNIR, respectively; both from Toptica Photonics AG, Gräfelfing, Germany) was used. A microscope objective (20× / N.A = 1.0) was used to focalize the laser excitation at a spot with a diameter (FWHM) ≈ 0.5 μm.

Cryosections were rehydrated in PBS and a coverslip was applied prior to imaging. CARS and SHG were acquired in transmission using pass-band emission filters with bands of 626–654 nm and 380–400 nm, respectively. TPEF was acquired in reflection using a bandpass filter of 500–550 nm. The three signals were encoded in RGB images for visualization purposes (red channel, CARS; green channel, TPEF; and blue channel, SHG).

The acquired field of view (FoV) was set to 152 μm × 302 μm. It was first scanned with a pixel size of 1 μm and pixel dwell of 3.4 µs (image acquisition time = 362 ms), then imaging was repeated with a pixel size of 0.2 μm and a 2-fold pixel averaging in order to obtain high-quality pictures for additional qualitative evaluation of the subcellular structures. In both cases, a tiling procedure was used to acquire multiple FoVs that covered a large sample area. Several single FoV images with CARS, TPEF, and SHG signals were acquired from each sample, and the actual number of images depended on the cryosection dimensions (mean = 418, SD = 155, median = 418, range: 80–941). Altogether, 64,414 single FoV images were acquired from all samples.

### Reference histology

After MPM imaging, all analyzed cryosections were briefly incubated in PBS to remove the coverslip and stained with hematoxylin and eosin (HE). All samples were analyzed by two experienced pathologists.

### Texture analysis and automated image classification

Images were exported from the microscope software as RGB images in TIFF format and original acquisition resolution. Before proceeding with the analysis, all single FoV images containing tissue borders, large holes, or other artefacts (e.g., dirt, air bubbles, artefacts due to the saturation of detectors) were discarded, as done in other experiments^31^. As a result, 55,282 of 64,414 images (86%) were passed to texture analysis and classification.

Texture analysis and automated classification of each FoV image were performed using MATLAB R2021b (The MathWorks Inc., Natick, MA, USA). Each image channel was analyzed separately. Therefore, after importing the TIFF files in MATLAB, the channels were split to obtain 8-bit single-channel images for CARS, TPEF and SHG. After min–max intensity normalization of each single-channel image, 17 texture parameters were calculated of each single-channel image using the built-in MATLAB functions. The first-order parameters included mean grey value, standard deviation, kurtosis, skewness, and entropy. The gray-level co-occurrence matrices were calculated for four orientations (0°, 45°, 90°, and 135°) and used to calculate the second-order parameters of contrast, correlation, energy, and homogeneity for three different distances (1, 12, and 30 μm). For each distance, texture parameters were obtained from the average of values calculated for the four orientations. The code for calculation of the texture parameters is reported in the Supplementary Information. The texture parameters were used for the classification of tumors versus liver tissue.

Based on the histopathological evaluation of HE-stained cryosections, two groups of tumor samples were identified, either entirely constituted by tumor tissue (*n* = 73) or containing the tumor border and regions of non-neoplastic liver tissue (*n* = 3). The latter patients were excluded from the dataset used to train the classifier and test its performance. Therefore, 35 randomly selected patients without tumor border in the tumor tissue sample were used to build the training set (patient data in Supplementary Table S1). The training set was composed of 25,298 images (12,647 images of liver tissue and 12,651 images of HCC), and the number of FoV images for each patient was highly variable (mean = 361, SD = 149, median = 349, range = 104–847), substantially depending on the tissue sample dimension. All other samples (*n* = 38) were used to test the classification (test set A; patient data in Supplementary Table S2). This test set was composed of 28,259 images (13,355 images of liver tissue and 14,904 images of HCC), with the number of FoV images in this case also variable for each patient (mean = 366, SD = 150, median = 350, range = 58–732).

The classification was performed using a neural network. The built-in MATLAB function “fitcnet” was used to train the neural network on the texture parameters. It trains a feedforward, fully connected neural network for classification. The weights of the neural network were initialized with randomized method using the default Glorot initializer to set the initial weights. A limited-memory Broyden-Fletcher-Goldfarb-Shanno quasi-Newton algorithm (L-BFGS) was employed as optimizer to train the model. The default loss function was cross-entropy, which was used to measure the train loss. The number of epochs used was 1000 (default value for the L-BFGS solver). The convergence criterium was based on reaching the iteration limit of 1000 epochs. In order to minimize losses, the neural network was trained using automatic Bayesian optimization of hyperparameters. The number of optimization steps was set to 100, and the acquisition function was set to “expected-improvement-plus” for better reproducibility.

Using the same training parameters, seven models were trained on the texture parameters of single-channel images as well as all their combinations, namely: CARS, TPEF, SHG, CARS-TPEF, CARS-SHG, TPEF-SHG, CARS-TPEF-SHG. The number of used texture parameters was 17 for the single-channel models, 34 for the two-channel models, and 51 for the three-channel model. The properties of the best models and training curves are provided in the Supplementary Information (Supplementary Tables S4 and Figure S1). Optimization histories are reported in Supporting Information as well. The models were then applied to provide classification scores (posterior probabilities) for each image of the independent test set using the built-in function “predict”. Assignment to the class “tumor” or “normal” for each image was made if the corresponding posterior probability exceeded 0.5.

The patients whose tumor samples included the tumor border (*n* = 3, patients’ data in Supplementary Table S3) were used as a second independent test set (test set B with 1,725 FoV images) to correlate the classification results with histological structures by creating maps of the posterior probability.

## Results

### Histopathological assessment of tissue architecture by label-free multiphoton microscopy

The evaluation of tissue morphology in both tumor and non-neoplastic background liver tissues was conducted using high-resolution images (0.2 μm/pixel) acquired with a tiling technique in order to visualize large regions. This approach facilitated simultaneous assessment of cell phenotypes and tissue architecture.

The analyzed cohort of tissue samples encompassed both HCC and non-neoplastic liver specimens, showing marked macroscopic heterogeneity. The samples exhibited conventional patterns associated with HCC^38^, as well as normal, steatotic, fibrotic, and cirrhotic features within the background liver.

**Fig. 1:**
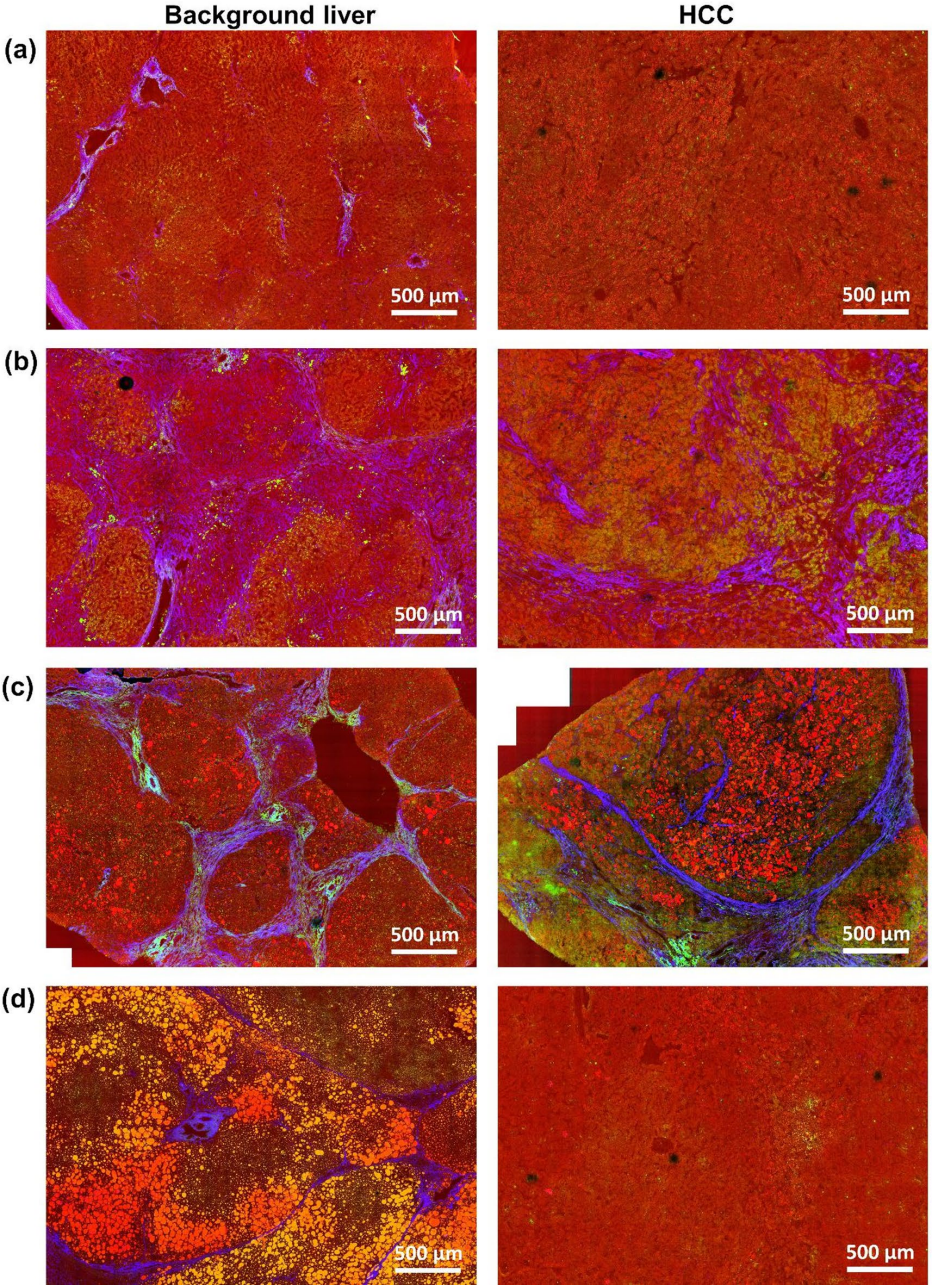
Label-free multimodal multiphoton images showing tissue architecture of background liver and HCC of four selected patients (red: CARS, green: TPEF, blue: SHG). (**a**) Uniform structure lacking fibrosis and fatty changes both in background liver and tumor tissue; (**b**) background liver tissue with cirrhotic nodules and tumor with fibrosis; (**c**) background liver tissue with cirrhotic nodules and fatty changes, tumor with fibrosis and fatty changes; (**d**) background liver tissue with marked fatty changes and fibrosis, tumor with homogenous structure lacking of both.

Figure 1 illustrates the macroscopic appearances of matched samples, presenting both background liver tissue and HCC of four representative patients (reference HE staining in Supplementary Fig. S2). Cellular structures are visualized through the combination of CARS (red) and TPEF (green), while fibrillar collagens are highlighted by SHG (blue). Elastin is detected using TPEF (green). The presence of lipid droplets is clearly indicated by a strong CARS (red) signal. Panel (a) shows normal liver parenchyma devoid of fibrosis and steatosis, along with a corresponding HCC exhibiting a relatively uniform structure, lacking fibrosis and fatty changes. While the SHG signal reveals collagen within the portal tracts of the normal liver, a notable aspect of tumor morphology is the absence of these tracts. Panels (b) and (c) show non-neoplastic liver tissue with characteristic cirrhotic nodules, and panel (c) shows additional fat accumulation. The corresponding HCC samples exhibit similar features. In panel (d), non-neoplastic liver tissue displays marked fatty changes and fibrosis, whereas the corresponding HCC presents ordinary histology without either of these features; the absence of portal tracts within the tumor is visible as well, and is confirmed by reference histology shown in Supplementary Fig. S2.

**Fig. 2:**
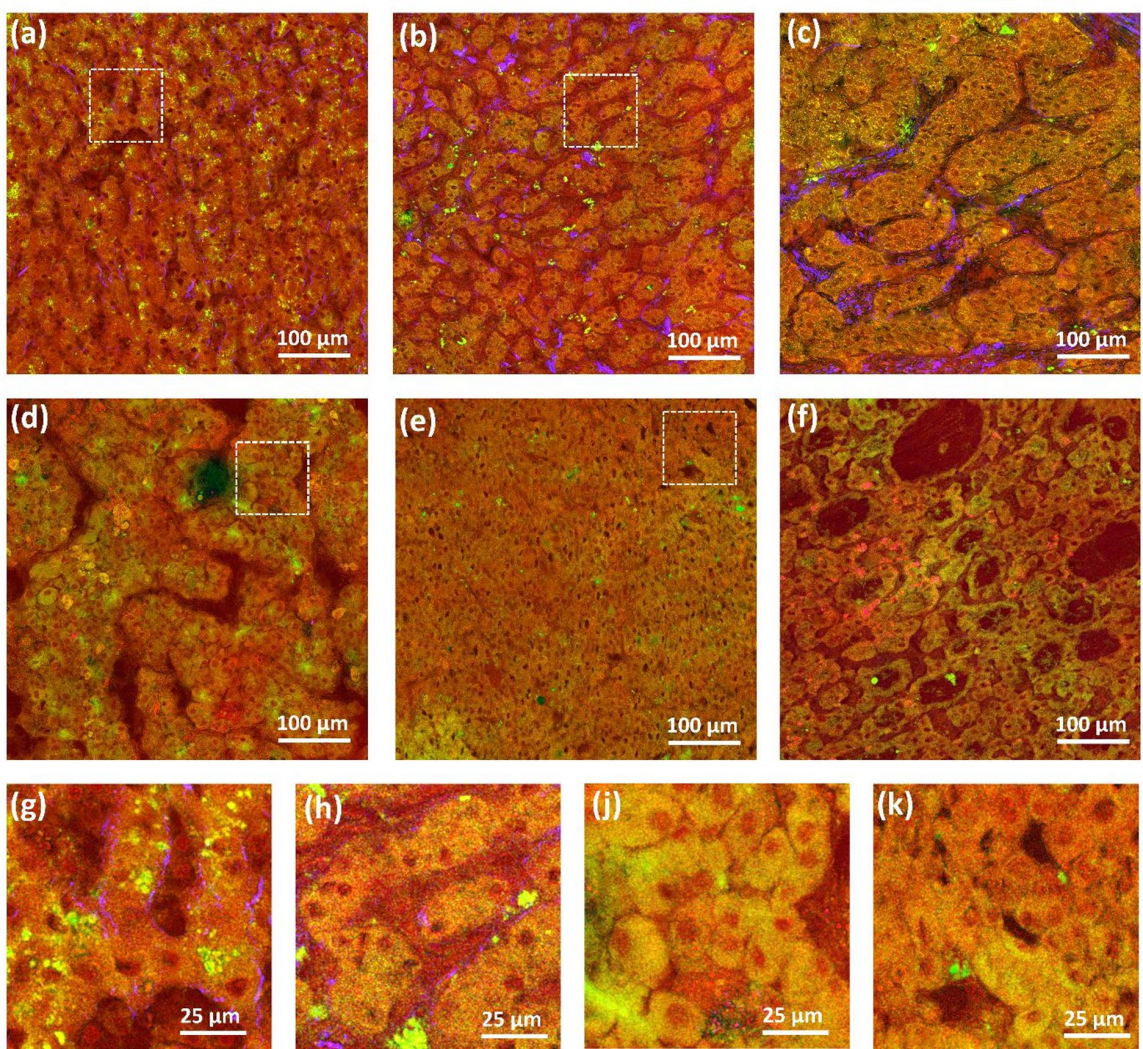
Label-free multimodal multiphoton images showing growth patterns of ordinary HCC at two different magnifications (red: CARS, green: TPEF, blue: SHG). (**a**,**g**) Normal liver parenchyma as reference; (**b**,**c**,**h**): trabecular; (**d**,**j**) macrotrabecular; (**e**,**k**) solid; (**f**) acinar.

The architectural features of ordinary HCC encompass trabecular, solid, acinar, and macrotrabecular (> 10 cells thick) patterns^39,40^, as shown in Fig. 2 (reference HE staining reported in Supplementary Fig. S3). Panel (a) displays the normal liver parenchyma as a reference. Panel (b) shows a well-differentiated HCC characterized by thin cell plates akin to normal liver tissue, with trabeculae consisting of two–three cells. In panel (c), a trabecular arrangement with thicker plates is visible, while panel (d) shows a macrotrabecular pattern with structure thickness exceeding ten cells. A tumor region with a solid growth pattern is displayed in panel (e). Acinar (pseudoglandular) structures are shown in panel (f). SHG imaging did not reveal any collagen fibers within the gaps between cell plates and acini, in line with the well-established understanding that these structures are not surrounded by stromal tissue^40^. The cell sizes in ordinary HCC were roughly equivalent to those of normal hepatocytes, occasionally slightly larger or smaller, as shown in the higher magnification images in panels (g-k) of Fig. 2.

**Fig. 3:**
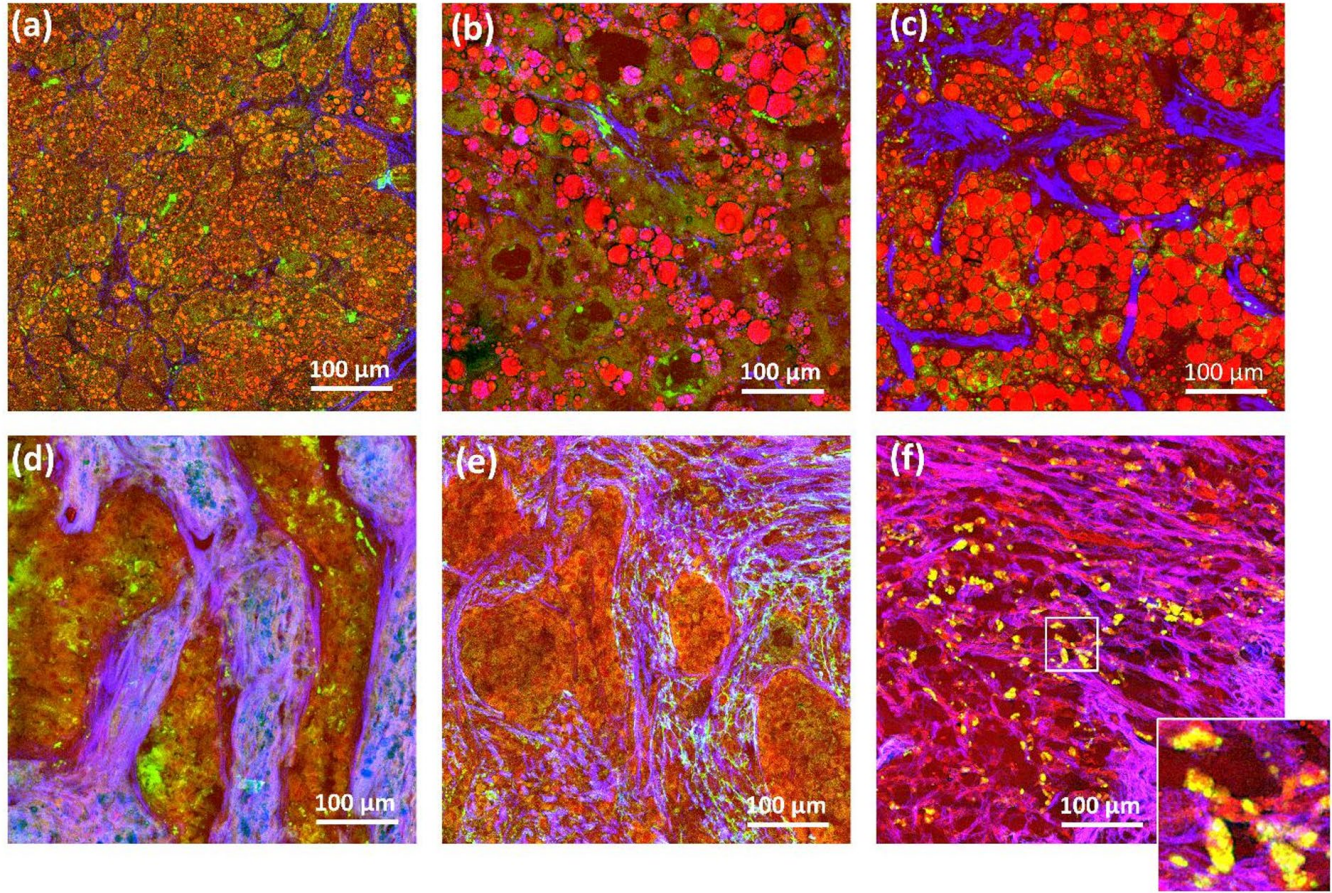
Label-free multiphoton images showing HCC samples with fatty changes and with increased stromal reactions. (**a**) Lipid droplets in steatohepatitic HCC; (**b**,**c**) large lipid droplets in steatotic HCC with fibrosis; (**d**,**e**) fibrotic changes; (**f**) extensive scarring with autofluorescent immune cells.

Fibrotic and fatty changes occurred to variable degrees in HCC. Lipid droplets were small, as in the case of steatohepatitic HCC shown in Fig. 3a, or macrovesicular, as in the steatotic HCCs in Fig. 3b,c. Reference histology is shown in Supplementary Fig. S4. The stroma within the tumor was usually not very prominent (see Fig. 2), but large bands of collagenous tissue were found to form distinctive nodularity, surrounding both large nodules (see Fig. 1) and smaller islands of neoplastic cells, as shown in Fig. 3d,e. Steatotic and steatohepatitic HCC often had intratumoral fibrosis, as visible in Fig. 3b,c. Scarring regions were also observed, as shown in Fig. 3f. Here, the strongly autofluorescent cells, shown in the inset, can be recognized as immune cells based on comparison with histological staining (Supplementary Fig. S4 (f)), in agreement with the results of previous studies^31^.

### Image classification

Image classification was performed using a neural network to distinguish between two groups: non-neoplastic background liver and tumor. The classification process relied on texture parameters extracted from the CARS, TPEF, and SHG channels within each Field of View (FoV) image. For each channel, a set of 17 texture parameters was calculated and utilized for classification.

**Fig. 4:**
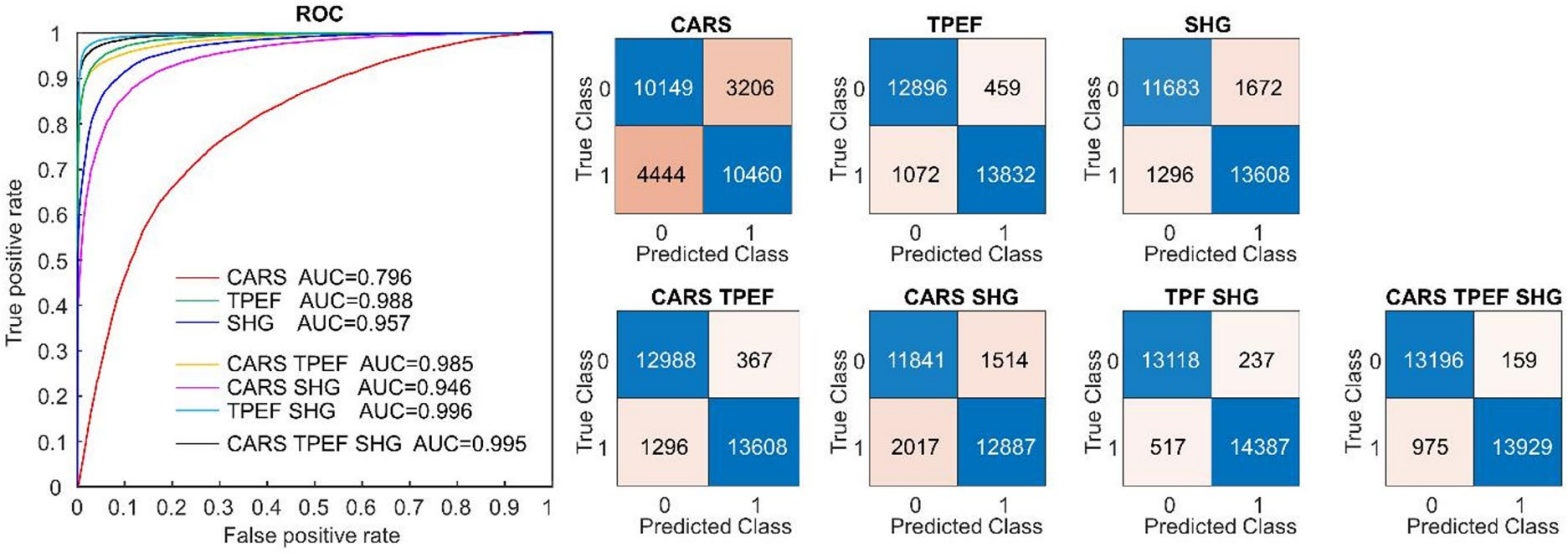
Classification performances of the test set with models based on different combinations of channels. ROC curves with AUC values of the different models and corresponding confusion matrices are shown (classes: 0 = liver tissue, 1 = tumor).

To evaluate the discriminative power of each nonlinear imaging modality, three distinct models were trained using the texture parameters of either CARS, TPEF, or SHG images. The performance of these models was assessed using the independent test set A of 38 patients. The Receiver Operating Characteristic (ROC) curves of the test set, along with the corresponding area under the curve (AUC) values, were calculated and are presented in Fig. 4, together with the confusion matrices. Notably, the classification based on CARS images exhibited a significantly lower accuracy compared to TPEF and SHG counterparts. The use of TPEF alone resulted in a correct classification rate of 94.6%.

Moreover, various combinations of texture parameters extracted from each signal were tested for classification to evaluate possible synergistic information. The ROC curves, AUC values, and confusion matrices of the test set classified with the models developed for all possible signal combinations are illustrated in Fig. 4. The best results emerged from the combination of TPEF and SHG, which achieved an overall correct classification rate of 97.3%. Interestingly, the different models demonstrated varying levels of sensitivity and accuracy, as shown by a comparison of the confusion matrices. For example, the TPEF-SHG-based model excelled in tumor recognition, while the three-channel model slightly outperformed in recognizing normal liver tissue.

Furthermore, the analysis of the posterior probability of class attribution obtained using the different models enabled retrieving the number of inconclusive attributions, which we defined as corresponding to a posterior probability between 0.3 and 0.7. The number of images with inconclusive classification was much larger for the CARS model (22.4%) in comparison to the SHG and TPEF models (8.9% and 4.9%, respectively), whereas it was very low for the TPEF-SHG and three-channel models (1.6% and 2.9%, respectively). This confirmed the optimal discriminative power of the latter two models as well as the pivotal role of TPEF. The frequency histograms are shown in Supplementary Fig. S5.

**Fig. 5:**
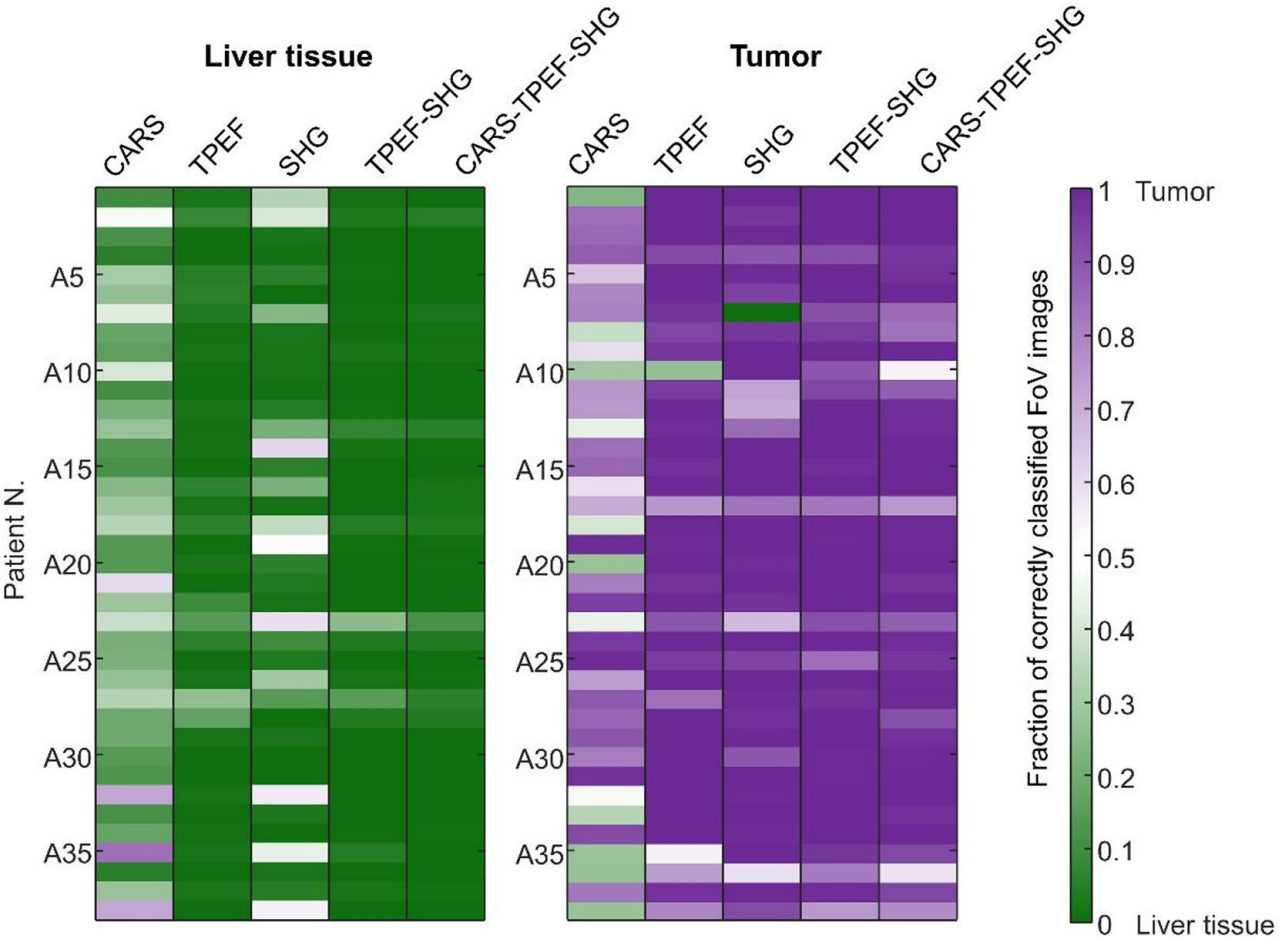
Classification of patients’ samples. Average classification probability for non-neoplastic liver and tumor samples of each patient in the test set A, calculated with selected classification models.

The classification results for each measured patient calculated as a fraction of the misclassified images are presented in Fig. 5 for selected models (CARS, TPEF, SHG, TPEF-SHG, and CARS-TPEF-SHG). Aligning with the statistics described above, the CARS-based model exhibited low accuracy and sensitivity across most patients irrespective of specific tissue features. Conversely, the TPEF-based model demonstrated higher accuracy, although it occasionally failed to identify the tumor tissue in certain patients. The TPEF-SHG-based model confirms a slightly better performance for recognition of tumor samples, while the CARS-TPEF-SHG-model for recognition of non-neoplastic samples.

The best classification model, TPEF-SHG, was also successful in recognizing well-differentiated G1 tumors. The correct classification rate of FoV images was 94%, only slightly lower than the correct rate attained for G2 tumor samples (96%). Unsurprisingly, the best correct rate was achieved for G3 tumors (99%). The correct rates of all classification models for different tumor grades are provided in Supplementary Table S5.

**Fig. 6:**
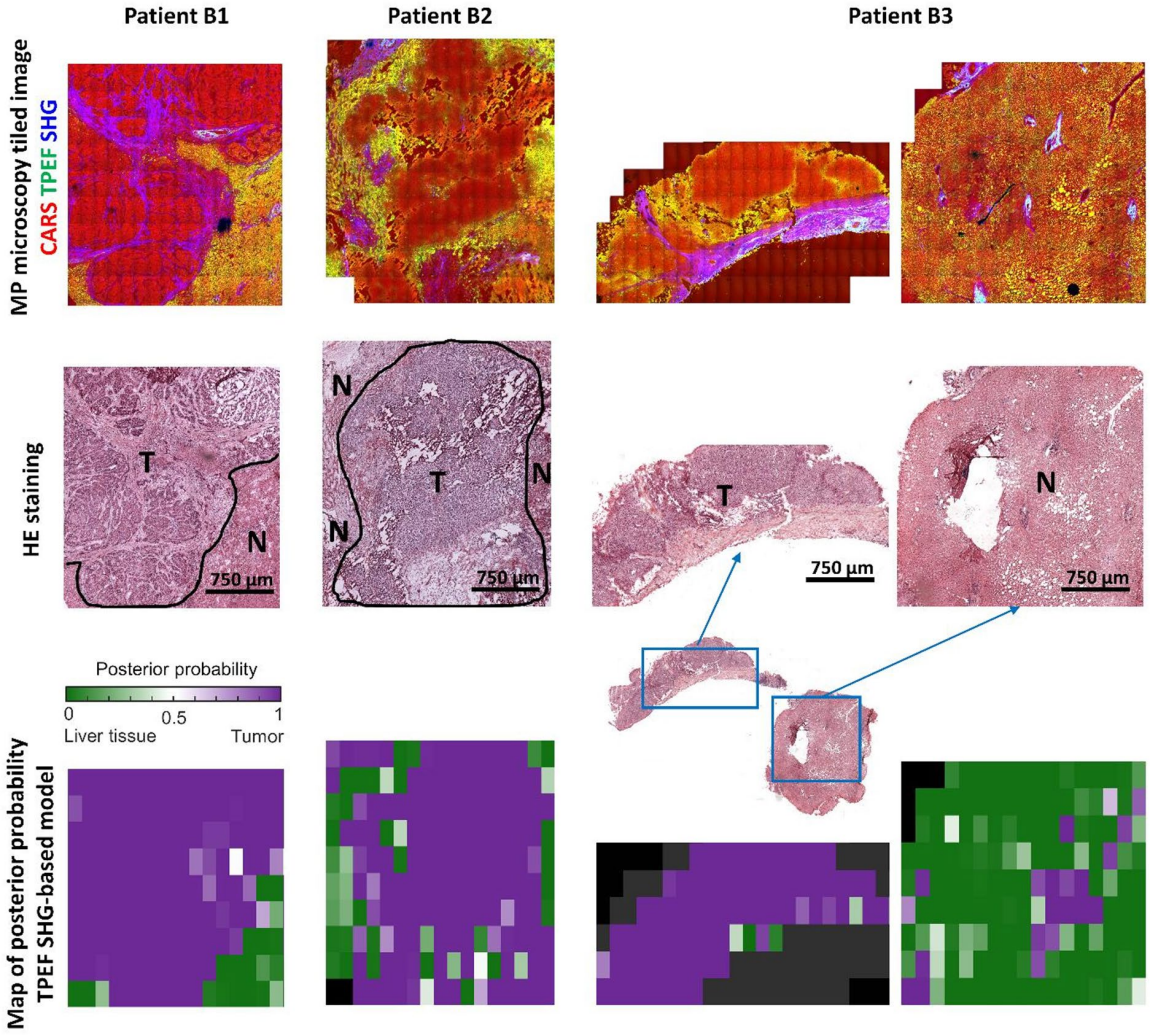
Maps of classification probability. Multiphoton images and corresponding histochemical staining of tumor samples containing the tumor border of the three patients in the test set B are reported for comparison with the classification maps calculated with the TPEF-SHG-based model. The localization of tumor borders is given for reference on the images of HE stained sections (T: tumor, N: non-neoplastic liver tissue). The tumor sample of patient B3 was separated in two parts, one of them being of normal tissue.

The TPEF-SHG model was finally applied to the texture parameter of images acquired from test set B of patients whose tumor samples contained the tumor border. The background liver tissue samples from these patients were classified correctly with a probability very close to or equal to one. Maps of the tumor samples were generated using the posterior probability of each FoV image and compared with the histological staining. As shown in Fig. 6, the regions of non-neoplastic liver tissue at the tumor borders were generally recognized correctly based on the comparison with histopathological tissue evaluation. Some cases of incorrect classification were observed in collagen-rich or steatotic regions.

## Discussion

The incidence of HCC has been constantly rising over the last decade, and now HCC accounts for the sixth most common cancer worldwide. Surgery remains the only curative treatment option with the lowest local recurrence rates. Complete tumor resection is essential for a successful oncological treatment. Tumor borders are often difficult to evaluate macroscopically because of frequently associated underlying liver diseases. Intraoperative frozen sections are the gold standard in cases of doubt regarding tumor-free resection margins. However, low-grade HCC (G1) is microscopically difficult to detect without specific staining or immunohistochemistry, and some resection margins remain unclear during surgery^5,6^. Other approaches have been tested, such as near-infrared fluorescence imaging. However, the sensitivity and specificity are comparably low^41^.

The use of intraoperative multiphoton microscopy in liver surgery is a new approach that aims to overcome these problems. We have already shown that MPM is suitable for the diagnosis of colorectal liver metastases^31^. In the current study, we addressed MPM and machine learning for HCC diagnosis and evaluation of resection margins.

Texture analysis is a fast and reliable method for extracting numerical parameters from single-channel images such as those generated by label-free MPM. We first successfully tested it for the classification of brain tumors by discriminant analysis^42^ and later applied the same approach for the recognition of colorectal liver metastases^31^. Discriminant analysis is a classification algorithm that is simple and fast to train, but is typically inferior in performance compared to deep-learning methods. For instance, the accuracy in recognizing non-neoplastic liver tissue remained limited to 95%, especially because the algorithm failed to classify correctly the regions with marked fibrosis^31^. Here, the texture parameters were fed to a neural network algorithm, which provided superior results and enabled to attain high correct rates for both HCC and non-neoplastic liver. Moreover, it was proven that a very reliable classification is achievable with 1 μm-resolution MPM images acquired at a frame rate of approximately 3 fps, which are qualitatively similar to the output of endoscopic multiphoton devices. Importantly, well-differentiated low-grade G1 tumors could be reliably distinguished from background liver tissue.

The acquisition of CARS, TPEF and SHG signals enabled the recapitulation of the most important features of both neoplastic and non-neoplastic liver tissue, so that recognition of microscopic tumor growth patterns as well as main pathological alterations of background liver tissue (steatosis and fibrosis) was possible from visual inspection of label-free MPM images. Although all three channels provide valuable histopathological information for the qualitative evaluation of tissue structures, their contributions to automated tissue classification are different. In particular, TPEF possesses very high power for discriminating HCC from the background liver tissue. Autofluorescence in the liver comes from different endogenous fluorophores dispersed inside the cell cytoplasm as well as within cell organelles, such as NAD(P)H, lipofuscin, and vitamin A^43^. As a result, TPEF images display, for example, cell density and provide indirect information about the dimension and form of the cell nuclei (which appear darker). Moreover, collagen possesses weak autofluorescence, whereas elastin possesses strong autofluorescence. TPEF also provides morphological information about lipid droplets, which are directly visible when they are autofluorescent (likely due to vitamin A^43^ or are indirectly displayed as dark holes when they lack fluorescence. Because of this somewhat unspecific but broad amount of information contained in TPEF images, a very good classification could be obtained based solely on the texture parameters of this channel. The inclusion of SHG in the analysis improved classification results. This is not surprising, as it is known that the patterns of collagen fibers, reticulin, and vascular phenotype are generally different between HCC and the background liver^44,45^. The information provided by CARS was not useful for further improving the classification performance.

This was a rather unexpected result, considering that the CARS images also showed cellular structures. Nevertheless, this knowledge is of practical importance for future exploitation of the method for intraoperative evaluation of liver tissue in situ during tumor resection. The generation of a CARS signal is only possible by using two ultrashort-pulsed laser beams that correctly overlap in the space and time domains. Although dedicated laser systems for multimodal CARS microscopy are found on the market, the realization of medical rigid endoscopes for simultaneous acquisition of CARS, TPEF, and SHG poses specific problems related to miniaturization, electrical safety, and optical quality (such as non-resonant background suppression)^34^. In contrast, a single laser beam is sufficient for the generation of TPEF and SHG, and this would significantly simplify both the required hardware and endoscope design. In principle, an endoscopic system designed only for autofluorescence imaging does not even require the incorporation of an ultrashort laser, as this can be excited by linear optical processes using short-wavelength light. Notably, spectral analysis of autofluorescence excited with UV or blue light and detected in the green-red spectral region enabled the identification of neoplastic tissue within the liver^43,46^. This opens up the possibility of using confocal laser endomicroscopes, which are already approved medical devices. They are normally used for fluorescent probe-based imaging (with fluorescein and indocyanine green, for instance)^47–49^, but initial feasibility studies have shown that they are sensitive enough to capture interpretable images of liver tissue autofluorescence when integrating a blue laser for excitation^49,50^.

Moving towards the deployment of label-free microscopic methods, some limitations shall be considered and eventually addressed in future research. These techniques are all characterized by a small penetration depth, meaning they can only provide surface information at the resection planes. There are clinical translation hurdles for prototype endoscopes. Sterility and mechanical robustness are nontrivial issues^51^. Endoscopic probes must be sterilized or use sterile sheaths. Deep learning for image classification shows promise, but the shift from the laboratory to the operating room must still be validated, which is currently a major bottleneck. It remains unclear whether models trained on ex vivo tissue can be generalized to in vivo intraoperative images due to artifacts and device differences. Intraoperative motion (e.g., respiration and heartbeat), the presence of blood, and surgical fluids may affect image quality and require rapid imaging or stabilization. Large quantities of blood in tissues, especially in the liver, might further decrease image quality due to light absorption. In the near future, large multicenter validation studies with prospective intraoperative image collection and synchronized histopathology will be necessary for regulatory approval. In this context, the explainability of classification models should also be considered, as black-box outputs are more difficult to validate clinically^52^.

In conclusion, label-free intraoperative optical histopathology is likely to play an important role in improving the tumor resection margins to ensure R0 resection in liver oncological surgery. Different imaging modalities might be endoscopically implemented to tailor on-site tissue analysis for optimization of tumor identification or characterization of background liver tissue. The presented approach based on multimodal images combined with deep-learning classification showed good performance on research datasets, but translation into reliable intraoperative tools still requires the availability of medical devices, large intraoperative datasets, and prospective clinical studies.

## Supplementary Information

Below is the link to the electronic supplementary material.

Supplementary Material 1

## Data Availability

The datasets used and analyzed in the current study are available from the corresponding author on reasonable request.
