# Supplementary material for: Label-free multiphoton microscopy and machine learning for recognition of hepatocellular carcinoma
Source: Sci Rep. 2026 Mar 10;16:8734. doi: 10.1038/s41598-026-43831-y (PMC12979795; doi:10.1038/s41598-026-43831-y)
Supplement: Supplementary file 1 — Supplementary Material 1 [file 41598_2026_43831_MOESM1_ESM.pdf]

Supplementary Information

Label-free multiphoton microscopy and machine learning for recognition of hepatocellular carcinoma

Roberta Galli [1,*], Sandra Korn [2], Daniela Aust [3,4], Gustavo B. Baretton [3,4], Jürgen Weitz [2,4], Edmund Koch [5], Carina Riediger [2,4]

[1] Department of Medical Physics and Biomedical Engineering, Faculty of Medicine Carl Gustav Carus, Technische Universität Dresden, D-01307 Dresden, Germany

[2] Department of Visceral, Thoracic and Vascular Surgery, University Hospital Carl Gustav Carus, Technische Universität Dresden, D-01307 Dresden, Germany

[3] Institute of Pathology, University Hospital Carl Gustav Carus, Medical Faculty, Technische Universität Dresden, D-01307 Dresden, Germany

[4] National Center for Tumor Diseases (NCT/UCC), Partner Site Dresden: German Cancer Research Center (DKFZ), D-69120 Heidelberg, Germany.

[5] Clinical Sensoring and Monitoring, Department of Anesthesiology and Intensive Care Medicine, Faculty of Medicine Carl Gustav Carus, Technische Universität Dresden, D-01307 Dresden, Germany

** corresponding author: roberta.galli@tu-dresden.de*

Table S1: Data of patients in the training set

N.	Age	Gender	BMI kg/m²	Diabetes [1]	Alcohol intake [2]	CHILD	Hepatitis	Chemo-therapy pre-OP [3]	Background liver pathologies [4]	Steato-hepatitis
1	20	f	19.6	no	0		no	no	3	
2	73	m	23.8	DM	0		no	no	1 3 4	
3	65	m	21.0	IDDM	0		no	no	0	
4	71	f	30.4	no	0		no	no	1 4	
5	79	m	30.0	IDDM	0		no	no	1 2 3	
6	69	m	29.4	DM	1		no	no	1 3	
7	85	m	24.9	DM	0		no	no	3	
8	59	m	35.9	DM	3	A	no	no	1 4	
9	45	f	21.0	IDDM	0		no	no	0	
10	77	m	24.2	IDDM	0	A	no	Yes (na)	1 2 4	CASH
11	66	f	33.1	DM	0	A	no	no	4 2	NASH
12	59	m	42.0	no	4	A	no	no	4 2	ASH
13	52	m	30.4	no	2		no	no	0	
14	73	m	28.1	IDDM	0	A	no	no	3	
15	70	m	21.8	no	4	A	no	no	4 6	
16	71	f	23.2	no	4	A	no	no	4 1	
17	54	m	24.8	no	3	A	no	yes (75)	4	
18	78	m	30.9	no	1	A	no	no	1 4	
19	81	m	29.3	DM	2		no	no	3 6	
20	75	m	29.0	no	0		no	no	1 3	
21	68	m	41.5	IDDM	0	A	no	no	4 1	
22	61	m	34.3	DM	5	A	no	no	4 3	
23	75	m	23.9	no	0	B	no	no	4	
24	72	m	21.1	no	3	B	no	no	4 1	
25	76	m	28.1	IDDM	0		no	no	2 1	
26	70	m	32.7	DM	0	A	no	no	4 6 2	NASH
27	56	m	18.5	DM	4	A	no	no	4 3 2	ASH
28	78	m	27.1	no	0	A	no	no	7 4 6	
29	73	m	29.8	no	2	A	no	no	3 4 6	
30	74	m	24.8	IDDM	1		no	no	1 3	
31	68	m	31.6	DM	0		no	no	1 3	
32	74	m	25.9	no	0		no	no	3	
33	77	m	28.1	DM	3	A	no	no	1 4	
34	70	f	18.4	no	2	A	no	no	1 3 4	
35	70	m	24.0	DM	0	A	no	no	4 7	

[1] DM: diabetes mellitus; IDDM: insulin-dependent diabetes mellitus

[2] 0: none; 1: moderate; 2: heavy; 3: alcoholic; 4: sober; 5: n.a.

[3] Number of days elapsed from last treatment until HCC surgery is given in parenthesis

[4] 0: none; 1: steatosis; 2: steatohepatitis; 3: fibrosis; 4: cirrhosis; 5: chronic hepatitis; 6: severe steatosis; 7: severe fibrosis.

Table S2: Data of patients in the test set A

N.	Age	Gender	BMI kg/m²	Diabetes (1)	Alcohol intake (2)	CHILD	Hepatitis	Chemo-therapy pre-OP	Background liver pathologies (3)	Steato-hepatitis
A1	77	m	25.2	IDDM	3		no	no	1 3	
A2	79	m	22.1	IDDM	3		no	no	1 3	
A3	77	m	35.8	IDDM	0	A	no	no	3 4	
A4	71	m	27.1	no	0		no	no	1	
A5	74	m	32.7	IDDM	4	A	no	no	1 4	
A6	72	m	30.0	no	0	A	no	yes (259)	3 4	
A7	70	m	33.1	no	0	A	no	no	1 4	
A8	59	m	35.9	DM	3	A	no	no	1 4	
A9	66	m	26.3	DM	0	A	no	no	4	
A10	72	f	31.2	no	0		no	no	3	
A11	77	m	33.6	IDDM	0		no	no	1 3	
A12	64	m	25.9	no	3	A	no	no	1 4	
A13	63	m	24.8	IDDM	2	A	no	no	4	
A14	64	m	28.1	DM	0	A	no	no	4	
A15	69	m	34.0	IDDM	5		no	no	1 4	
A16	73	m	22.7	no	0		no	no	3	
A17	68	m	33.7	IDDM	1	A	no	no	4	
A18	66	m	31.4	IDDM	0	A	no	no	4 1	
A19	81	m	33.3	DM	0		Hep B	no	3	
A20	67	m	23.6	IDDM	4		no	no		
A21	60	m	44.2	no	0		no	no	3 2 6	
A22	78	f	23.6	IDDM	0	A	no	no	7	
A23	56	f	33.0	IDDM	3	B	no	n.a.	4 1 7 2	ASH
A24	73	m	26.8	DM	3	A	no	no	1 4	
A25	62	m	23.9	DM	0	A	no	no	4	
A26	62	m	29.8	no	3	A	no	no	6 4 2	ASH
A27	67	m	25.1	no	1	A	no	no	4 2	NASH
A28	60	m	31.6	DM	2		no	no	6 3	
A29	70	m	25.8	DM	0		no	no	1	
A30	77	m	23.7	no	0		no	no	1 3	
A31	78	m	28.7	DM	0		no	no	6 3	
A32	66	m	26.8	DM	2		no	no	3 6	
A33	69	f	30.2	no	0		no	no	1 3	
A34	77	m	33.3	DM	2		no	no	1 3	
A35	68	m	35.2	DM	0	A	no	no	4	
A36	79	f	21.1	no	0		no	no	3 6	
A37	70	f	37.0	no	0		no	no	3 6	
A38	67	m	26.8	DM	2		no	no	3 6	

Table S3: Data of patients in the test set B (tumor tissue samples with tumor border)

N.	Age	Gender	BMI kg/m²	Diabetes (1)	Alcohol intake (2)	CHILD	Hepatitis	Chemo-therapy pre-OP	Background liver pathologies (3)	Steato-hepatitis
B1	77	m	31.9	no	0		no	no	3	
B2	79	m	28.9	IDDM	0		no	no		
B3	70	f	37.0	no	0		no	no	1 2	NASH

Matlab code for calculation of texture parameters based on use of built-in Matlab functions

```
function param = texture_parameters(A);


% FIRST-ORDER PARAMETERS

% A is the matrix of the 8-bit single-channel image (CARS, TPEF or SHG).

% mean
meangray=mean(A);

% standard deviation
SDgray=std(A);

%kurtosis
kurt=kurtosis(A);

% skewness
skew=skewness(A);

% entropy
entrop=entropy(A);

% SECOND-ORDER PARAMETERS
% gray-level co-occurrence matrices are calculated for 4 orientation (0°,45°, 90° and 135°) and for two
different offsets (given by D1 and D2).

D1=1;   % range on "near-field" texture (number of pixels)

glcm_near_0= graycomatrix(A,'Offset',[0 D1]);
glcm_near_45= graycomatrix(A,'Offset',[-D1 D1]);
glcm_near_90= graycomatrix(A,'Offset',[-D1 0]);
glcm_near_135= graycomatrix(A,'Offset',[-D1 -D1]);

D2=12;   % range on "mid-field" texture (number of pixels)

glcm_mid_0= graycomatrix(A,'Offset',[0 D2]);
glcm_mid_45= graycomatrix(A,'Offset',[-D2 D2]);
glcm_mid_90= graycomatrix(A,'Offset',[-D2 0]);
glcm_mid_135= graycomatrix(A,'Offset',[-D2 -D2]);

D3=30;   % range on "far-field" texture (number of pixels)

glcm_far_0= graycomatrix(A,'Offset',[0 D3]);
glcm_far_45= graycomatrix(A,'Offset',[-D3 D3]);
glcm_far_90= graycomatrix(A,'Offset',[-D3 0]);
glcm_far_135= graycomatrix(A,'Offset',[-D3 -D3]);

stats_near_0 = graycoprops(glcm_near_0,'Contrast Correlation Energy Homogeneity');
stats_near_45 = graycoprops(glcm_near_45,'Contrast Correlation Energy Homogeneity');
stats_near_90 = graycoprops(glcm_near_90,'Contrast Correlation Energy Homogeneity');
stats_near_135 = graycoprops(glcm_near_135,'Contrast Correlation Energy Homogeneity');

stats_mid_0 = graycoprops(glcm_mid_0,'Contrast Correlation Energy Homogeneity');
stats_mid_45 = graycoprops(glcm_mid_45,'Contrast Correlation Energy Homogeneity');
stats_mid_90 = graycoprops(glcm_mid_90,'Contrast Correlation Energy Homogeneity');
stats_mid_135 = graycoprops(glcm_mid_135,'Contrast Correlation Energy Homogeneity');

stats_far_0 = graycoprops(glcm_far_0,'Contrast Correlation Energy Homogeneity');
stats_far_45 = graycoprops(glcm_far_45,'Contrast Correlation Energy Homogeneity');
stats_far_90 = graycoprops(glcm_far_90,'Contrast Correlation Energy Homogeneity');
stats_far_135 = graycoprops(glcm_far_135,'Contrast Correlation Energy Homogeneity');

% the parameters with offset D1, D2 and D3 are calculated as averages on the four directions.

contr_near=(stats_near_0.Contrast+stats_near_45.Contrast+stats_near_90.Contrast+stats_near_135.Contrast
)/4;
contr_mid=(stats_mid_0.Contrast+stats_mid_45.Contrast+stats_mid_90.Contrast+stats_mid_135.Contrast)/4;
contr_far=(stats_far_0.Contrast+stats_far_45.Contrast+stats_far_90.Contrast+stats_far_135.Contrast)/4;
```

```
corr_near=(stats_near_0.Correlation+stats_near_45.Correlation+stats_near_90.Correlation+stats_near_135.
Correlation)/4;
corr_mid=(stats_mid_0.Correlation+stats_mid_45.Correlation+stats_mid_90.Correlation+stats_mid_135.Corre
lation)/4;
corr_far=(stats_far_0.Correlation+stats_far_45.Correlation+stats_far_90.Correlation+stats_far_135.Corre
lation)/4;

energy_near=(stats_near_0.Energy+stats_near_45.Energy+stats_near_90.Energy+stats_near_135.Energy)/4;
energy_mid=(stats_mid_0.Energy+stats_mid_45.Energy+stats_mid_90.Energy+stats_mid_135.Energy)/4;
energy_far=(stats_far_0.Energy+stats_far_45.Energy+stats_far_90.Energy+stats_far_135.Energy)/4;

homog_near=(stats_near_0.Homogeneity+stats_near_45.Homogeneity+stats_near_90.Homogeneity+stats_near_135
.Homogeneity)/4;
homog_mid=(stats_mid_0.Homogeneity+stats_mid_45.Homogeneity+stats_mid_90.Homogeneity+stats_mid_135.Homo
geneity)/4;
homog_far=(stats_far_0.Homogeneity+stats_far_45.Homogeneity+stats_far_90.Homogeneity+stats_far_135.Homo
geneity)/4;


% output vector with 17 texture parameters

param = ([meangray SDgray kurt skew entrop contr_near contr_mid contr_far corr_near corr_mid corr_far
energy_near energy_mid energy_far homog_near homog_mid homog_far])';
```

Table S4: Classification model properties.

	CARS	TPEF	SHG	CARS-TPEF	CARS-SHG	TPEF-SHG	CATS-TPEF-SHG
**Activations**	tanh	tanh	tanh	relu	sigmoid	relu	relu
**Standardize**	true	true	true	true	true	true	true
**Lambda***	1.9155e-05	1.0868e-04	6.9867e-06	2.0172e-04	2.2444e-05	1.2655e-05	1.2748e-06
**LayerSizes**	[100, 267]	[292, 60, 207]	[289, 218, 11]	[253, 10, 35]	[298, 12]	[280, 210]	[283, 6, 186]
**Observed objective function value**	0.10736	0.017669	0.045458	0.0090126	0.030912	0.010475	0.0061665
**Estimated objective function value**	0.10944	0.017664	0.046977	0.0095214	0.032694	0.010421	0.0062205

* Lambda: regularization strength

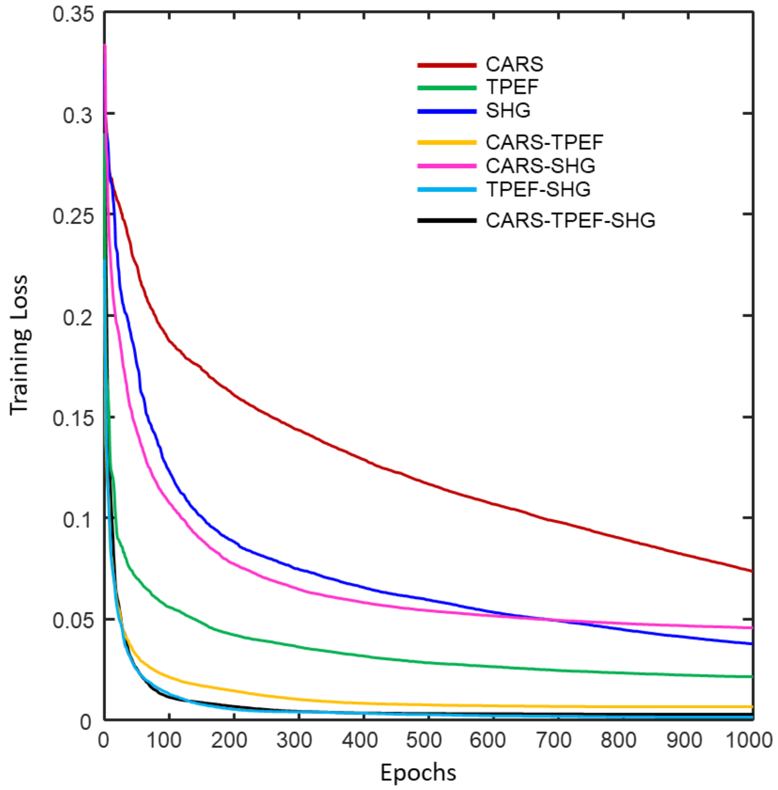

Supporting Figure S1: Training histories.

Optimization histories

CARS model

Iter	Eval result	Objective	Objective runtime	BestSoFar (observed)	BestSoFar (estim)	Activations	Standardize	Lambda	LayerSizes
1	Best	0.25022	860.93	0.25022	0.25022	relu	false	4.01E-05	[291 35]
2	Best	0.21567	47.839	0.21567	0.21704	sigmoid	true	2.83E-08	[2 6 8]
3	Best	0.11333	916.49	0.11333	0.1242	tanh	true	3.41E-09	[59 264]
4	Accept	0.21836	22.915	0.11333	0.12116	relu	true	2.28E-05	2
5	Best	0.11112	744.37	0.11112	0.11131	tanh	true	6.01E-09	[39 249]
6	Accept	0.28939	42.543	0.11112	0.11116	tanh	true	0.034328	[1 81]
7	Accept	0.23595	29.015	0.11112	0.11115	tanh	true	9.81E-08	[1 4]
8	Accept	0.12989	369.97	0.11112	0.11117	tanh	true	2.42E-09	[10 137]
9	Accept	0.11701	1216.9	0.11112	0.11122	tanh	true	8.67E-08	[116 294]
10	Accept	0.21646	654.09	0.11112	0.11113	tanh	true	1.99E-08	[2 276]
11	Accept	0.1255	89.801	0.11112	0.11114	tanh	true	1.88E-09	[30 1]
12	Best	0.11009	306.48	0.11009	0.11019	tanh	true	1.16E-09	[43 73 1]
13	Accept	0.11432	626.84	0.11009	0.11019	tanh	true	6.61E-10	[31 217 2]
14	Accept	0.11108	720.82	0.11009	0.11022	tanh	true	4.73E-07	[54 28 210]
15	Accept	0.11697	772.59	0.11009	0.11022	tanh	true	1.44E-08	289
16	Accept	0.25061	561.64	0.11009	0.11014	tanh	true	0.0064728	293
17	Accept	0.11653	247.81	0.11009	0.1102	tanh	true	3.60E-08	[83 7]
18	Accept	0.11819	2327.5	0.11009	0.11022	tanh	true	4.03E-10	[273 296 142]
19	Accept	0.11108	376.68	0.11009	0.11075	tanh	true	6.14E-08	[28 60 57]
20	Accept	0.11795	970.58	0.11009	0.1111	tanh	true	1.68E-08	[294 51 17]
21	Accept	0.11131	866.22	0.11009	0.1106	tanh	true	2.54E-07	[40 292 7]
22	Accept	0.11839	736.48	0.11009	0.1117	tanh	true	6.13E-08	[47 185 47]
23	Accept	0.11934	541.19	0.11009	0.11193	tanh	true	4.34E-10	[103 90]
24	Accept	0.1242	105.08	0.11009	0.11149	tanh	true	5.15E-07	36
25	Accept	0.11131	783.64	0.11009	0.11122	tanh	true	3.05E-08	[38 265]
26	Accept	0.11562	769.43	0.11009	0.1115	tanh	true	7.48E-09	[22 284]
27	Accept	0.11104	691.52	0.11009	0.11177	tanh	true	5.27E-08	[36 237]
28	Accept	0.1142	795.62	0.11009	0.11022	tanh	true	5.59E-10	[300 2]
29	Accept	0.11803	1226.1	0.11009	0.11174	tanh	true	1.20E-09	[253 1 279]
30	Accept	0.1125	785.87	0.11009	0.11186	tanh	true	3.37E-09	[39 286 1]
31	Accept	0.11618	358.05	0.11009	0.11189	tanh	true	1.03E-07	[36 97]
32	Accept	0.11123	867.6	0.11009	0.11171	tanh	true	1.42E-09	[42 298]
33	Accept	0.22638	945.55	0.11009	0.11169	tanh	false	4.03E-10	[262 58 3]
34	Accept	0.24567	23.16	0.11009	0.11166	none	true	3.70E-08	[2 8 11]
35	Accept	0.30769	115.26	0.11009	0.11166	sigmoid	false	2.74E-09	[4 21 23]
36	Accept	0.49992	23.122	0.11009	0.1118	none	false	3.8003	298
37	Accept	0.49992	7.5837	0.11009	0.11174	sigmoid	true	0.82221	[269 130 4]
38	Accept	0.49992	5.0657	0.11009	0.11176	relu	true	3.4694	[293 8]
39	Accept	0.40276	32.315	0.11009	0.11177	tanh	false	4.82E-08	[1 15]
40	Accept	0.49992	3.6376	0.11009	0.11179	none	true	2.2167	257
41	Accept	0.41446	78.981	0.11009	0.11181	relu	false	1.20E-08	[1 65]
42	Accept	0.49992	27.747	0.11009	0.11183	tanh	false	3.7403	288
43	Accept	0.34438	20.299	0.11009	0.11181	relu	true	4.38E-10	[1 3]
44	Accept	0.49992	1.3614	0.11009	0.11162	relu	true	3.1728	[1 72]
45	Best	0.10985	1654.4	0.10985	0.11115	tanh	true	2.12E-06	[240 109 254]
46	Accept	0.49992	3.293	0.10985	0.11174	tanh	true	0.42964	[133 2 3]
47	Best	0.10748	716.19	0.10748	0.10757	tanh	true	2.73E-05	[50 228]
48	Accept	0.11337	1676.4	0.10748	0.10767	tanh	true	2.59E-05	[262 269]
49	Accept	0.11621	697.95	0.10748	0.10767	sigmoid	true	5.00E-10	295
50	Accept	0.121	569.32	0.10748	0.10767	sigmoid	true	3.99E-10	[34 11 222]
51	Accept	0.11384	1127.2	0.10748	0.10768	sigmoid	true	1.20E-08	[172 255]
52	Accept	0.49992	36.352	0.10748	0.10766	relu	false	3.4671	[292 76 245]
53	Accept	0.11606	220.93	0.10748	0.10768	sigmoid	true	2.71E-09	91
54	Accept	0.24219	164.6	0.10748	0.10768	none	true	4.09E-10	[291 2 74]
55	Accept	0.12171	631.82	0.10748	0.10767	relu	true	5.13E-07	289
56	Accept	0.1223	662.88	0.10748	0.1077	relu	true	1.33E-08	[287 18 2]
57	Accept	0.12183	803.19	0.10748	0.10771	relu	true	1.94E-07	[49 265 42]
58	Accept	0.49992	12.966	0.10748	0.1077	sigmoid	false	0.13481	[265 37 32]
59	Accept	0.11301	1119.4	0.10748	0.11185	tanh	true	3.41E-06	[98 299]
60	Accept	0.17507	581.14	0.10748	0.11196	tanh	true	4.69E-05	[4 238 3]
61	Accept	0.11661	1279.8	0.10748	0.11218	sigmoid	true	1.45E-09	[228 226 40]
62	Accept	0.11732	637.33	0.10748	0.11215	sigmoid	true	6.43E-07	270
63	Accept	0.11961	684.25	0.10748	0.11224	sigmoid	true	5.88E-08	292
64	Accept	0.11301	366.31	0.10748	0.11223	sigmoid	true	5.47E-07	[64 89]
65	Accept	0.2867	31.515	0.10748	0.11223	none	false	5.37E-10	[1 4]
66	Accept	0.11942	376.35	0.10748	0.11228	sigmoid	true	1.46E-07	[95 62]
67	Accept	0.1206	120.44	0.10748	0.11226	sigmoid	true	1.04E-05	48
68	Accept	0.25939	582.16	0.10748	0.11227	none	false	5.09E-10	[293 4]
69	Accept	0.10819	565.24	0.10748	0.10796	tanh	true	2.94E-05	[96 106 5]
70	Accept	0.11408	1125.7	0.10748	0.10796	sigmoid	true	3.08E-06	[135 298]
71	Accept	0.12523	443.43	0.10748	0.10799	relu	true	9.86E-08	204

72	Accept	0.12483	1006.2	0.10748	0.10804	relu	true	4.24E-10	[290 3 182]
73	Accept	0.12349	719.92	0.10748	0.10807	relu	true	6.28E-06	[90 224 7]
74	Accept	0.4511	175.91	0.10748	0.10799	relu	false	4.19E-10	[295 34 5]
75	Accept	0.23974	138.92	0.10748	0.10798	sigmoid	true	0.00016355	[1 62]
76	Accept	0.26176	27.615	0.10748	0.10798	none	true	4.12E-10	[1 3]
77	Accept	0.11119	1373.5	0.10748	0.1087	tanh	true	1.90E-05	[74 282 130]
78	Accept	0.11819	185.35	0.10748	0.10868	sigmoid	true	1.24E-06	76
79	Best	0.10736	1021.9	0.10736	0.10812	tanh	true	1.92E-05	[100 267]
80	Accept	0.26943	406.93	0.10736	0.10813	none	false	1.64E-05	[1 202 6]
81	Accept	0.11499	792.37	0.10736	0.10811	sigmoid	true	4.04E-10	[108 207]
82	Accept	0.10807	827.59	0.10736	0.10803	tanh	true	1.58E-05	[120 191 1]
83	Accept	0.10973	728.73	0.10736	0.10826	tanh	true	2.60E-05	[94 135 44]
84	Accept	0.1104	978.12	0.10736	0.10836	tanh	true	3.90E-05	[82 277 4]
85	Accept	0.11444	325.96	0.10736	0.10856	tanh	true	1.56E-05	120
86	Accept	0.11266	1337.3	0.10736	0.10905	tanh	true	1.12E-05	[86 268 111]
87	Accept	0.13207	114.41	0.10736	0.10902	relu	true	1.60E-06	49
88	Accept	0.11179	1008.9	0.10736	0.10924	tanh	true	3.45E-05	[86 290 2]
89	Accept	0.11803	1953.1	0.10736	0.10941	tanh	true	4.48E-06	[239 293 89]
90	Accept	0.11704	1229.7	0.10736	0.10943	relu	true	2.30E-05	[283 188]
91	Accept	0.11878	994.36	0.10736	0.10944	relu	true	2.78E-09	[80 165 176]
92	Accept	0.12096	1560.2	0.10736	0.10945	relu	true	4.09E-06	[282 286]
93	Accept	0.25994	510.87	0.10736	0.10944	none	false	1.98E-06	256
94	Accept	0.49992	0.50317	0.10736	0.10944	sigmoid	false	3.8225	[1 18 5]
95	Accept	0.24563	1158	0.10736	0.10944	sigmoid	false	4.03E-10	[299 108 35]
96	Accept	0.49992	0.50594	0.10736	0.10944	relu	false	2.312	1
97	Accept	0.49992	3.1354	0.10736	0.10944	tanh	false	3.8961	[1 169]
98	Accept	0.49992	0.3792	0.10736	0.10944	none	true	3.8566	[1 13]
99	Accept	0.22037	854.3	0.10736	0.10944	tanh	false	4.92E-05	[298 7]
100	Accept	0.23077	828.81	0.10736	0.10944	tanh	false	1.83E-07	293

TPEF model

Iter	Eval result	Objective	Objective runtime	BestSoFar (observed)	BestSoFar (estim)	Activations	Standardize	Lambda	LayerSizes
1	Best	0.077832	315.37	0.077832	0.077832	none	false	2.76E-06	158
2	Accept	0.49992	1.8528	0.077832	0.10198	sigmoid	true	0.024106	[6 1 30]
3	Accept	0.080599	792.22	0.077832	0.089239	relu	false	0.00090016	[260 38]
4	Accept	0.083564	308.13	0.077832	0.077871	tanh	false	1.66E-09	[4 91]
5	Best	0.076409	386.19	0.076409	0.076656	sigmoid	false	2.67E-06	156
6	Best	0.021543	381.08	0.021543	0.021557	sigmoid	true	3.63E-06	162
7	Accept	0.077437	361.42	0.021543	0.021555	sigmoid	false	2.75E-06	[137 2]
8	Accept	0.058858	180.2	0.021543	0.021554	none	true	3.44E-06	112
9	Accept	0.057475	146.85	0.021543	0.021553	tanh	true	3.85E-07	[1 58]
10	Accept	0.022373	504.45	0.021543	0.021556	sigmoid	true	3.82E-06	[175 38 2]
11	Accept	0.057356	20.303	0.021543	0.02159	sigmoid	true	5.72E-08	1
12	Accept	0.49992	1.8515	0.021543	0.021551	sigmoid	true	3.9409	[69 22]
13	Accept	0.043679	857.44	0.021543	0.021554	tanh	true	0.0015628	[3 188 118]
14	Accept	0.49992	0.52838	0.021543	0.021533	tanh	true	3.94	[2 6]
15	Best	0.019013	499.08	0.019013	0.019005	tanh	true	4.42E-05	[171 8]
16	Accept	0.058858	169.66	0.019013	0.019004	none	true	2.04E-09	[97 174]
17	Accept	0.16472	25.719	0.019013	0.019005	sigmoid	false	8.27E-10	2
18	Accept	0.083169	206.25	0.019013	0.019005	none	false	6.48E-10	[3 97]
19	Accept	0.10206	3.1079	0.019013	0.019004	none	true	0.014983	35
20	Accept	0.41719	165.78	0.019013	0.019006	relu	false	1.56E-07	[128 125 1]
21	Accept	0.20069	102.37	0.019013	0.019008	relu	false	0.39521	287
22	Accept	0.076607	865.2	0.019013	0.019008	tanh	false	2.56E-06	[64 234]
23	Accept	0.10198	434.03	0.019013	0.019008	none	false	0.0074631	[4 207 5]
24	Accept	0.10317	466.58	0.019013	0.019008	tanh	false	0.0078438	[2 152]
25	Accept	0.028145	259.5	0.019013	0.019011	tanh	true	3.96E-10	93
26	Accept	0.49992	10.256	0.019013	0.019012	sigmoid	false	0.030715	[154 84 2]
27	Accept	0.025298	74.123	0.019013	0.019012	relu	true	3.82E-06	[10 23]
28	Accept	0.024468	170.02	0.019013	0.019011	relu	true	2.41E-08	[21 57]
29	Accept	0.037276	643.94	0.019013	0.01901	relu	true	0.0028813	[13 284 3]
30	Accept	0.49992	4.8357	0.019013	0.019014	relu	true	2.9278	254
31	Accept	0.021029	617.84	0.019013	0.019012	relu	true	0.00015806	[200 72]
32	Accept	0.078109	220.94	0.019013	0.019011	relu	true	4.18E-10	[1 52 21]
33	Accept	0.49992	1.6635	0.019013	0.019012	none	false	3.8803	[25 1]
34	Accept	0.081785	62.263	0.019013	0.019011	none	false	0.00019248	[21 5]
35	Accept	0.49992	0.96722	0.019013	0.019013	tanh	false	3.9457	[25 15]
36	Accept	0.078979	157.35	0.019013	0.019012	tanh	false	0.00018746	[3 34 8]
37	Accept	0.058858	99.945	0.019013	0.01901	none	true	7.49E-08	189
38	Accept	0.024271	225.23	0.019013	0.019012	tanh	true	5.99E-09	[47 31]
39	Accept	0.49992	4.0879	0.019013	0.019012	none	true	3.9287	264
40	Accept	0.060677	14.585	0.019013	0.019012	none	true	0.00034334	8
41	Accept	0.082022	68.412	0.019013	0.019011	none	false	3.41E-08	[19 7]
42	Accept	0.023322	601.7	0.019013	0.019011	sigmoid	true	7.68E-07	251

43	Accept	0.02767	303.78	0.019013	0.01901	sigmoid	true	3.95E-10	125
44	Accept	0.051546	131.78	0.019013	0.019011	relu	true	2.59E-07	[2 48]
45	Accept	0.081232	48.561	0.019013	0.019011	sigmoid	false	1.10E-07	9
46	Accept	0.020871	773.72	0.019013	0.019091	tanh	true	0.00021348	[28 293]
47	Accept	0.076172	210.2	0.019013	0.019096	tanh	false	5.36E-08	[19 5 45]
48	Accept	0.021346	199.27	0.019013	0.019103	relu	true	2.18E-05	86
49	Accept	0.041821	747.95	0.019013	0.019092	relu	true	6.06E-09	[3 44 291]
50	Accept	0.3222	98.173	0.019013	0.018999	relu	true	0.00022518	[1 2 101]
51	Accept	0.020871	541.93	0.019013	0.018999	tanh	true	6.71E-06	[21 56 127]
52	Accept	0.084394	1448.9	0.019013	0.019001	none	false	5.65E-05	[16 279 260]
53	Accept	0.021069	779.96	0.019013	0.019005	tanh	true	1.21E-09	[53 3 265]
54	Accept	0.022848	1728.9	0.019013	0.019031	sigmoid	true	4.64E-10	[31 277 288]
55	Accept	0.023243	1370.5	0.019013	0.019008	relu	true	1.40E-06	[293 222]
56	Accept	0.058898	872.21	0.019013	0.019007	none	true	2.79E-07	[74 123 288]
57	Accept	0.058937	704.07	0.019013	0.019039	none	true	0.00023585	[7 97 287]
58	Accept	0.057396	22.574	0.019013	0.019002	tanh	true	4.58E-10	1
59	Accept	0.023282	1297.5	0.019013	0.019002	tanh	true	6.66E-08	[206 49 235]
60	Accept	0.082931	770.64	0.019013	0.019035	sigmoid	false	4.44E-07	[5 4 257]
61	Accept	0.059926	294.04	0.019013	0.01901	relu	true	0.0028195	140
62	Accept	0.021622	905.03	0.019013	0.019011	sigmoid	true	5.33E-07	[119 3 288]
63	Best	0.017669	1428.9	0.017669	0.017641	tanh	true	0.00010868	[292 60 207]
64	Accept	0.058819	151.49	0.017669	0.015915	none	true	4.33E-10	[142 2 121]
65	Accept	0.089889	792.86	0.017669	0.01764	none	false	5.06E-09	[4 85 281]
66	Accept	0.021859	989.91	0.017669	0.017638	sigmoid	true	7.27E-09	[199 6 231]
67	Accept	0.057752	142.97	0.017669	0.017676	tanh	true	0.0001353	[1 1 54]
68	Accept	0.098111	596.48	0.017669	0.017675	relu	false	0.0061575	[5 11 207]
69	Accept	0.02178	1478.7	0.017669	0.017679	tanh	true	4.62E-10	[261 223 18]
70	Accept	0.069215	2579.4	0.017669	0.017678	tanh	false	4.84E-10	[269 195 287]
71	Accept	0.037869	701.33	0.017669	0.01925	relu	true	0.0021799	[66 257]
72	Accept	0.024073	1407.6	0.017669	0.017669	relu	true	1.39E-05	[262 260]
73	Accept	0.059056	160.49	0.017669	0.016086	relu	true	0.00074054	[1 110]
74	Accept	0.021504	1390.7	0.017669	0.016016	tanh	true	4.16E-09	[283 218 19]
75	Accept	0.024508	641.79	0.017669	0.015977	relu	true	7.38E-09	285
76	Accept	0.022018	1617.7	0.017669	0.015949	tanh	true	1.04E-05	[253 274 2]
77	Accept	0.022808	1100.9	0.017669	0.018585	tanh	true	1.07E-06	[244 8 184]
78	Accept	0.039924	664.82	0.017669	0.015976	tanh	true	0.00065022	276
79	Accept	0.047593	541.55	0.017669	0.015863	sigmoid	true	1.42E-09	[2 225 18]
80	Accept	0.02265	1303.8	0.017669	0.018999	sigmoid	true	7.78E-10	[277 2 287]
81	Accept	0.058028	226.46	0.017669	0.018461	sigmoid	true	1.13E-06	[1 89 16]
82	Accept	0.023243	902.16	0.017669	0.018453	sigmoid	true	6.16E-08	[278 1 122]
83	Accept	0.072298	311.81	0.017669	0.018163	none	true	7.36E-09	[1 6 244]
84	Accept	0.025733	561.67	0.017669	0.015642	relu	true	9.46E-09	[246 2 5]
85	Accept	0.020397	1875.4	0.017669	0.017692	tanh	true	0.00011417	[295 287]
86	Accept	0.21302	292.07	0.017669	0.011832	relu	true	0.0013701	[240 1 1]
87	Accept	0.023875	612.98	0.017669	0.012579	relu	true	1.29E-06	274
88	Accept	0.023678	746.69	0.017669	0.012301	tanh	true	3.76E-06	276
89	Accept	0.021148	538.16	0.017669	0.011837	tanh	true	3.21E-09	[210 1 1]
90	Accept	0.043521	1271.2	0.017669	0.017653	relu	true	0.003347	[236 290]
91	Accept	0.02514	425.82	0.017669	0.017657	sigmoid	true	4.15E-10	[111 88]
92	Accept	0.02435	1441.5	0.017669	0.015688	sigmoid	true	1.71E-07	[267 246]
93	Accept	0.027196	750.56	0.017669	0.017652	tanh	true	1.80E-08	294
94	Accept	0.080757	28.453	0.017669	0.015604	tanh	false	2.30E-05	1
95	Accept	0.022018	668.71	0.017669	0.017657	sigmoid	true	8.31E-07	[293 1 1]
96	Accept	0.073207	750.33	0.017669	0.017658	tanh	false	4.02E-10	270
97	Accept	0.084513	582.88	0.017669	0.015648	none	false	4.15E-10	[241 1 81]
98	Accept	0.025694	1449.7	0.017669	0.015661	relu	true	6.31E-09	[280 286]
99	Accept	0.023599	1402.6	0.017669	0.017659	relu	true	9.20E-06	[241 281]
100	Accept	0.024034	543.64	0.017669	0.017664	relu	true	4.77E-09	[245 13]

SHG model

Iter	Eval result	Objective	Objective runtime	BestSoFar (observed)	BestSoFar (estim)	Activations	Standardize	Lambda	LayerSizes
1	Best	0.17128	327.37	0.17128	0.17128	sigmoid	false	4.60E-10	[71 62 1]
2	Best	0.063048	644.27	0.063048	0.092189	sigmoid	true	5.48E-09	[18 264]
3	Accept	0.49992	14.903	0.063048	0.063094	relu	false	0.20177	[6 137]
4	Accept	0.49992	4.8321	0.063048	0.063107	sigmoid	false	0.54577	58
5	Accept	0.087635	70.556	0.063048	0.068168	sigmoid	true	8.39E-10	[4 24]
6	Accept	0.49992	6.8204	0.063048	0.063132	sigmoid	true	2.3036	[281 45]
7	Best	0.056882	404.57	0.056882	0.056943	sigmoid	true	3.10E-07	[32 144]
8	Accept	0.18053	139.49	0.056882	0.056942	sigmoid	false	2.70E-07	[41 1 15]
9	Accept	0.49992	2.0681	0.056882	0.05694	sigmoid	false	3.948	[83 11 17]
10	Accept	0.21614	54.76	0.056882	0.056935	none	true	4.29E-10	[43 9 2]
11	Accept	0.27231	22.009	0.056882	0.056934	none	true	0.011187	[26 125]
12	Accept	0.25781	28.075	0.056882	0.056931	none	false	4.12E-10	2
13	Accept	0.36529	18.242	0.056882	0.056932	none	false	0.48304	184

14	Accept	0.057238	368.77	0.056882	0.056845	sigmoid	true	5.76E-08	154
15	Accept	0.20638	498.35	0.056882	0.05687	sigmoid	true	7.19E-08	[1 175 44]
16	Accept	0.15946	925.32	0.056882	0.056985	sigmoid	false	4.10E-10	[159 18 229]
17	Accept	0.0604	846.64	0.056882	0.057025	sigmoid	true	4.04E-10	[61 89 205]
18	Accept	0.061744	332.25	0.056882	0.057108	sigmoid	true	4.92E-08	[55 3 89]
19	Best	0.052415	391.18	0.052415	0.052429	sigmoid	true	1.52E-06	[159 7]
20	Accept	0.055933	1317.4	0.052415	0.052512	sigmoid	true	3.35E-07	[247 243]
21	Best	0.050439	790.73	0.050439	0.050466	tanh	true	4.09E-10	[196 85]
22	Accept	0.079848	595.75	0.050439	0.050462	tanh	true	1.44E-09	[4 166 64]
23	Best	0.048423	1351.4	0.048423	0.048438	tanh	true	2.21E-07	[243 142 79]
24	Accept	0.087161	38.401	0.048423	0.048427	tanh	true	7.70E-05	[4 3]
25	Accept	0.056724	143.48	0.048423	0.048459	tanh	true	3.11E-08	49
26	Accept	0.2235	1293.6	0.048423	0.048484	tanh	true	0.010849	[295 184]
27	Accept	0.050992	1436.2	0.048423	0.048535	tanh	true	9.68E-09	[299 5 271]
28	Accept	0.21168	952.55	0.048423	0.048471	tanh	true	4.48E-07	[1 92 283]
29	Accept	0.055894	109.61	0.048423	0.048467	tanh	true	4.17E-10	[34 2]
30	Accept	0.048937	468.98	0.048423	0.048442	tanh	true	7.50E-06	[78 90]
31	Accept	0.12653	723.4	0.048423	0.048438	tanh	false	4.35E-10	282
32	Accept	0.33675	19.758	0.048423	0.048441	tanh	false	1.38E-07	[1 2]
33	Accept	0.059451	857	0.048423	0.048438	relu	true	4.45E-10	[281 77]
34	Accept	0.10321	25.061	0.048423	0.048435	relu	true	1.59E-09	4
35	Accept	0.06044	1582.8	0.048423	0.048438	relu	true	2.92E-07	[296 284]
36	Accept	0.17144	27.071	0.048423	0.048438	relu	true	0.00019683	[5 4 1]
37	Accept	0.05704	317.57	0.048423	0.048439	relu	true	1.15E-08	[120 26]
38	Accept	0.49992	4.891	0.048423	0.048437	relu	true	3.6974	260
39	Accept	0.059096	609.94	0.048423	0.048437	relu	true	3.41E-07	[13 13 263]
40	Accept	0.21678	21.745	0.048423	0.048452	relu	true	4.78E-07	1
41	Accept	0.14796	217.22	0.048423	0.048452	relu	true	1.14E-06	[55 67 1]
42	Accept	0.059886	1659.9	0.048423	0.049187	relu	true	5.77E-08	[289 159 213]
43	Accept	0.055143	708.43	0.048423	0.04936	relu	true	4.63E-10	[28 297]
44	Accept	0.055182	333.11	0.048423	0.048451	tanh	true	7.91E-06	[19 105]
45	Accept	0.49992	0.68534	0.048423	0.048465	tanh	true	0.92523	[4 26]
46	Accept	0.055498	648.03	0.048423	0.048465	relu	true	8.95E-10	[37 45 225]
47	Accept	0.059649	834.04	0.048423	0.046634	tanh	true	2.06E-07	[297 2]
48	Best	0.048383	816.47	0.048383	0.048532	tanh	true	2.34E-05	[97 30 192]
49	Accept	0.072693	47.563	0.048383	0.048523	relu	true	1.32E-08	16
50	Accept	0.12127	812.63	0.048383	0.048527	tanh	false	1.43E-05	[293 16]
51	Accept	0.49992	25.931	0.048383	0.04737	tanh	false	2.463	283
52	Accept	0.12373	843.58	0.048383	0.048527	tanh	false	7.90E-08	290
53	Accept	0.049213	1621.7	0.048383	0.048512	tanh	true	7.61E-09	[138 113 269]
54	Accept	0.060005	725.18	0.048383	0.048511	sigmoid	true	5.02E-10	297
55	Accept	0.26255	584.13	0.048383	0.048406	none	true	1.84E-08	[1 2 271]
56	Accept	0.055222	442.85	0.048383	0.048433	relu	true	2.15E-08	[17 15 180]
57	Accept	0.053482	363	0.048383	0.048431	tanh	true	1.14E-06	[48 4 89]
58	Best	0.048028	1559.4	0.048028	0.047987	tanh	true	4.62E-06	[204 80 280]
59	Accept	0.060874	501.61	0.048028	0.047642	relu	true	8.28E-09	220
60	Accept	0.063009	235.97	0.048028	0.047645	relu	true	4.77E-10	[93 2]
61	Accept	0.49992	6.9737	0.048028	0.04734	relu	false	6.05E-10	[259 145 2]
62	Accept	0.054668	287.41	0.048028	0.047359	tanh	true	6.09E-09	[17 92 1]
63	Accept	0.24939	612.36	0.048028	0.048006	none	false	4.22E-10	[285 33]
64	Accept	0.33228	468.93	0.048028	0.047375	sigmoid	false	2.44E-09	[1 2 211]
65	Accept	0.056605	607.78	0.048028	0.047403	sigmoid	true	6.75E-06	[48 119 88]
66	Accept	0.056091	955.6	0.048028	0.047668	sigmoid	true	9.76E-07	[77 300 3]
67	Accept	0.053759	925.57	0.048028	0.048052	tanh	true	1.41E-06	[115 215 1]
68	Accept	0.050281	2204.3	0.048028	0.04807	tanh	true	4.17E-10	[269 213 208]
69	Accept	0.066329	870.62	0.048028	0.049399	relu	true	9.09E-08	[8 134 227]
70	Accept	0.049253	165.44	0.048028	0.048372	tanh	true	4.81E-05	[27 9 27]
71	Accept	0.053048	736.98	0.048028	0.049658	tanh	true	1.12E-05	[42 11 254]
72	Accept	0.051071	454.16	0.048028	0.049853	tanh	true	1.35E-09	[124 39 2]
73	Accept	0.054352	186.12	0.048028	0.048037	tanh	true	4.26E-10	[11 35 22]
74	Accept	0.05708	1022.4	0.048028	0.049412	tanh	true	2.29E-07	[130 2 275]
75	Accept	0.065499	53.079	0.048028	0.049399	sigmoid	true	4.28E-10	17
76	Accept	0.064432	1897.5	0.048028	0.049383	sigmoid	true	5.04E-09	[289 249 110]
77	Accept	0.25065	179.63	0.048028	0.049386	none	false	0.00035096	[1 91]
78	Accept	0.049016	1910.3	0.048028	0.048679	tanh	true	2.93E-09	[245 249 118]
79	Best	0.047276	1811.8	0.047276	0.047981	tanh	true	8.00E-06	[246 282 53]
80	Accept	0.2163	1246.2	0.047276	0.047473	none	true	1.31E-06	[299 260]
81	Accept	0.056842	754.89	0.047276	0.047412	tanh	true	3.68E-05	[48 244]
82	Accept	0.058858	667.65	0.047276	0.047414	sigmoid	true	1.43E-05	282
83	Accept	0.055538	230.9	0.047276	0.04718	sigmoid	true	4.53E-06	96
84	Accept	0.06044	891.42	0.047276	0.04682	sigmoid	true	7.41E-06	[174 2 211]
85	Accept	0.055024	686.31	0.047276	0.047483	tanh	true	4.22E-05	[13 243 21]
86	Accept	0.054194	468.28	0.047276	0.048022	tanh	true	7.26E-06	[137 3 25]
87	Accept	0.069057	51.593	0.047276	0.047985	sigmoid	true	7.30E-06	[15 1]
88	Accept	0.056289	1266.4	0.047276	0.048978	tanh	true	3.62E-09	[29 230 189]
89	Accept	0.047909	1474	0.047276	0.046889	tanh	true	1.78E-05	[291 170]
90	Best	0.045458	1646.6	0.045458	0.046313	tanh	true	6.99E-06	[289 218 11]
91	Accept	0.49992	0.3861	0.045458	0.046324	none	true	3.5625	[1 2]

92	Accept	0.15412	147.74	0.045458	0.046342	tanh	false	4.09E-10	[24 27]
93	Accept	0.49992	3.9159	0.045458	0.046371	none	true	3.7196	297
94	Accept	0.057356	698.52	0.045458	0.046326	sigmoid	true	1.62E-06	293
95	Accept	0.049095	1603.1	0.045458	0.046207	tanh	true	4.94E-06	[289 213]
96	Accept	0.050874	3160.5	0.045458	0.046403	tanh	true	1.03E-06	[287 265 291]
97	Accept	0.058937	620.18	0.045458	0.046313	tanh	true	7.24E-08	[24 219 2]
98	Accept	0.49992	7.4984	0.045458	0.046564	none	false	3.8986	[1 212]
99	Accept	0.24733	100.98	0.045458	0.046338	none	false	2.03E-06	47
100	Accept	0.051506	1359.7	0.045458	0.046438	tanh	true	2.15E-08	[184 266]

CARS-TPEF model

Iter	Eval result	Objective	Objective runtime	BestSoFar (observed)	BestSoFar (estim)	Activations	Standardize	Lambda	LayerSizes
1	Best	0.056526	959.99	0.056526	0.056526	relu	false	4.01E-05	[291 35]
2	Best	0.03103	69.029	0.03103	0.032044	sigmoid	true	2.83E-08	[2 6 8]
3	Best	0.014823	320.97	0.014823	0.014837	tanh	true	3.41E-09	[59 264]
4	Accept	0.031742	34.831	0.014823	0.014931	relu	true	2.28E-05	2
5	Accept	0.03439	185.87	0.014823	0.015324	tanh	true	0.0028681	[4 66]
6	Best	0.014428	49.539	0.014428	0.014462	tanh	true	9.11E-09	56
7	Best	0.013242	225.98	0.013242	0.01326	tanh	true	4.58E-09	[35 24 186]
8	Accept	0.041466	54.395	0.013242	0.013245	tanh	true	2.18E-09	[1 12]
9	Accept	0.49992	6.7238	0.013242	0.013248	sigmoid	true	0.25412	116
10	Accept	0.12594	43.399	0.013242	0.013249	relu	true	1.94E-07	[3 3 1]
11	Accept	0.044905	837.19	0.013242	0.013248	tanh	false	1.27E-07	[300 9]
12	Accept	0.12373	1072.1	0.013242	0.013248	none	false	7.09E-06	[1 192 253]
13	Accept	0.24207	506.94	0.013242	0.013251	tanh	false	2.83E-05	[1 2 277]
14	Best	0.010712	222	0.010712	0.010779	relu	true	1.71E-09	[294 94]
15	Accept	0.013637	438.49	0.010712	0.010724	tanh	true	2.17E-05	[13 1 174]
16	Accept	0.49992	5.367	0.010712	0.010724	tanh	true	0.40316	[226 1 1]
17	Accept	0.49992	5.9498	0.010712	0.010734	sigmoid	true	0.044019	[2 1 94]
18	Accept	0.042493	523.76	0.010712	0.011681	tanh	true	0.00026572	[1 232]
19	Accept	0.024073	177	0.010712	0.010841	tanh	true	1.31E-06	[3 61]
20	Accept	0.030121	92.564	0.010712	0.010841	sigmoid	true	5.25E-08	[2 29 3]
21	Accept	0.02099	774.92	0.010712	0.010838	tanh	true	0.0034941	[273 9 48]
22	Accept	0.072614	1048	0.010712	0.01084	none	false	3.28E-06	[186 264 9]
23	Accept	0.077437	171.01	0.010712	0.010843	none	false	9.13E-07	[3 77 1]
24	Accept	0.49992	4.5749	0.010712	0.010841	relu	true	0.69508	[210 2 45]
25	Accept	0.043245	239.27	0.010712	0.010839	none	true	3.64E-08	[6 203 1]
26	Accept	0.04281	263.09	0.010712	0.01084	none	true	3.25E-06	[9 145 7]
27	Accept	0.043363	471.37	0.010712	0.010839	none	true	7.38E-07	[61 148 73]
28	Accept	0.043205	445.28	0.010712	0.010838	none	true	1.28E-08	[1 87 282]
29	Accept	0.059017	164.4	0.010712	0.010839	tanh	false	1.82E-08	52
30	Accept	0.066567	56.939	0.010712	0.01084	relu	false	2.78E-06	8
31	Accept	0.010989	722.26	0.010712	0.010836	sigmoid	true	2.19E-05	[300 7]
32	Accept	0.052494	788.78	0.010712	0.010837	sigmoid	false	2.70E-06	[297 4]
33	Accept	0.043521	395.63	0.010712	0.010836	none	true	0.00077848	[293 70]
34	Accept	0.072693	927.81	0.010712	0.017527	none	false	7.96E-05	[297 117]
35	Accept	0.10194	48.526	0.010712	0.010842	none	false	0.020851	16
36	Accept	0.23753	44.169	0.010712	0.010849	sigmoid	false	0.00071074	[14 1 1]
37	Accept	0.49992	0.61593	0.010712	0.010845	relu	false	0.1273	[1 8 1]
38	Accept	0.3273	14.349	0.010712	0.01085	sigmoid	false	6.58E-10	1
39	Accept	0.048304	9.2221	0.010712	0.01085	none	true	0.00085583	1
40	Accept	0.49992	80.428	0.010712	0.010847	sigmoid	false	0.080467	[125 253 278]
41	Accept	0.050043	1564.1	0.010712	0.010736	tanh	false	1.20E-06	[226 258 1]
42	Accept	0.10194	628.9	0.010712	0.010929	none	false	1.10E-09	[280 89]
43	Accept	0.49992	0.65383	0.010712	0.01074	tanh	false	3.7319	[3 11 6]
44	Accept	0.49992	3.9331	0.010712	0.010741	none	true	1.3002	[272 3]
45	Accept	0.012452	576.79	0.010712	0.010741	sigmoid	true	2.47E-06	[239 2]
46	Accept	0.024192	95.697	0.010712	0.010768	relu	true	4.07E-10	[4 34]
47	Accept	0.056012	744.26	0.010712	0.010767	relu	false	3.98E-10	268
48	Accept	0.013005	598.9	0.010712	0.010769	sigmoid	true	4.05E-10	[282 28 202]
49	Accept	0.043205	64.274	0.010712	0.010771	none	true	4.04E-10	[48 12]
50	Accept	0.49992	2.6815	0.010712	0.010771	none	false	3.9321	17
51	Accept	0.056012	652.82	0.010712	0.010773	relu	false	1.49E-08	239
52	Accept	0.049055	861.74	0.010712	0.010774	tanh	false	3.97E-10	[289 6]
53	Accept	0.043007	479.58	0.010712	0.010776	none	true	8.62E-05	[268 29 7]
54	Accept	0.041901	32.669	0.010712	0.018065	sigmoid	true	8.45E-06	[1 2]
55	Accept	0.014033	158.25	0.010712	0.01078	tanh	true	5.85E-08	227
56	Accept	0.011819	854.75	0.010712	0.010743	tanh	true	4.32E-06	[298 1 49]
57	Accept	0.071745	49.727	0.010712	0.010781	none	false	0.0013787	[1 7 3]
58	Accept	0.07214	578.31	0.010712	0.010782	none	false	4.01E-08	296
59	Best	0.0090126	614.7	0.0090126	0.009051	relu	true	0.00020172	[253 10 35]
60	Accept	0.042533	21.763	0.0090126	0.0091482	relu	true	0.0001825	1
61	Accept	0.0098031	874.35	0.0090126	0.0094053	relu	true	5.41E-05	[295 77]
62	Accept	0.015416	695.64	0.0090126	0.009422	sigmoid	true	2.66E-09	[296 262 91]

63	Accept	0.012886	981.64	0.0090126	0.0094436	tanh	true	0.0012496	[295 2 115]
64	Accept	0.041505	27.077	0.0090126	0.0094494	sigmoid	true	6.55E-10	1
65	Accept	0.012175	599.78	0.0090126	0.0094549	sigmoid	true	7.60E-08	[290 29]
66	Accept	0.012056	251.77	0.0090126	0.0094449	relu	true	4.02E-10	[285 4 63]
67	Accept	0.012689	766.97	0.0090126	0.0094367	tanh	true	4.13E-10	[294 1 300]
68	Accept	0.32801	17.298	0.0090126	0.0093681	sigmoid	false	2.44E-07	[1 3]
69	Accept	0.051625	710.79	0.0090126	0.0093323	sigmoid	false	1.71E-05	267
70	Accept	0.010475	749.71	0.0090126	0.0093396	tanh	true	4.86E-05	[293 1 2]
71	Accept	0.012175	791.86	0.0090126	0.009346	sigmoid	true	4.09E-07	[293 8 29]
72	Accept	0.012531	299.3	0.0090126	0.0093308	tanh	true	1.18E-08	[291 38 2]
73	Accept	0.012768	1093.5	0.0090126	0.0093357	tanh	true	2.42E-07	[296 101]
74	Accept	0.011621	1058.2	0.0090126	0.0093427	sigmoid	true	8.10E-06	[262 145]
75	Accept	0.011938	803.79	0.0090126	0.0093352	tanh	true	1.69E-05	[296 3]
76	Accept	0.41786	53.693	0.0090126	0.009146	relu	false	2.16E-09	[1 85]
77	Accept	0.056052	843.39	0.0090126	0.0091264	relu	false	1.32E-06	[298 5]
78	Accept	0.3275	15.281	0.0090126	0.0091125	tanh	false	4.04E-10	1
79	Accept	0.095383	37.421	0.0090126	0.0091128	none	false	1.68E-09	1
80	Accept	0.011819	145.04	0.0090126	0.0091448	relu	true	0.00010545	[37 26 1]
81	Accept	0.045537	832.27	0.0090126	0.009142	tanh	false	4.46E-09	299
82	Accept	0.049727	409.62	0.0090126	0.0091429	none	true	4.20E-10	[1 152 106]
83	Accept	0.043245	487.3	0.0090126	0.0091448	none	true	5.66E-09	[300 143]
84	Accept	0.011345	408.5	0.0090126	0.0091457	tanh	true	0.00083508	[37 113 20]
85	Accept	0.071547	99.067	0.0090126	0.0091471	relu	false	5.23E-05	[1 30]
86	Accept	0.012649	74.833	0.0090126	0.0091478	sigmoid	true	1.03E-06	22
87	Accept	0.075737	583.87	0.0090126	0.0091492	none	false	0.0020649	291
88	Accept	0.043047	171.86	0.0090126	0.0091512	none	true	6.55E-05	[1 2 98]
89	Accept	0.013519	124.18	0.0090126	0.0091528	relu	true	8.56E-10	[70 3]
90	Accept	0.013519	299.9	0.0090126	0.009154	tanh	true	4.54E-10	[26 68 167]
91	Accept	0.012452	175.02	0.0090126	0.0091549	tanh	true	1.59E-06	[51 6]
92	Accept	0.016484	50.755	0.0090126	0.0091557	sigmoid	true	8.22E-10	[37 8]
93	Accept	0.0092893	906.54	0.0090126	0.0084732	relu	true	0.00010629	[288 95]
94	Accept	0.0098822	1562.1	0.0090126	0.0089391	relu	true	4.96E-06	[297 144 196]
95	Accept	0.010317	644.73	0.0090126	0.008893	relu	true	1.38E-05	[286 3]
96	Accept	0.01174	169.69	0.0090126	0.0088193	relu	true	5.40E-08	[279 12]
97	Accept	0.0097241	1327.2	0.0090126	0.0088135	relu	true	5.42E-07	[297 207]
98	Accept	0.013045	139.24	0.0090126	0.0088251	sigmoid	true	1.29E-07	53
99	Accept	0.011661	165.26	0.0090126	0.0088322	relu	true	8.08E-09	265
100	Accept	0.011345	143.42	0.0090126	0.0088096	sigmoid	true	1.17E-05	[49 4]

CARS-SHG model

Iter	Eval result	Objective	Objective runtime	BestSoFar (observed)	BestSoFar (estim)	Activations	Standardize	Lambda	LayerSizes
1	Best	0.12807	838.45	0.12807	0.12807	relu	false	4.01E-05	[291 35]
2	Best	0.11886	60.744	0.11886	0.11923	sigmoid	true	2.83E-08	[2 6 8]
3	Best	0.038699	889.95	0.038699	0.046344	tanh	true	3.41E-09	[59 264]
4	Accept	0.11768	34.24	0.038699	0.044117	relu	true	2.28E-05	2
5	Accept	0.039608	870.43	0.038699	0.038929	tanh	true	2.01E-08	[67 249]
6	Accept	0.056882	50.487	0.038699	0.038846	tanh	true	5.61E-10	[5 3]
7	Best	0.034351	109.78	0.034351	0.034428	tanh	true	0.00063623	[32 2]
8	Accept	0.038027	99.195	0.034351	0.034438	tanh	true	4.88E-10	[24 5]
9	Accept	0.49992	1.1773	0.034351	0.034374	tanh	true	2.7416	[32 9]
10	Accept	0.038382	1196.5	0.034351	0.034385	tanh	true	1.22E-05	[290 116 2]
11	Accept	0.1493	271.7	0.034351	0.034392	tanh	true	7.46E-05	[1 112]
12	Accept	0.039015	819.29	0.034351	0.034398	tanh	true	0.00025574	295
13	Best	0.03186	717.53	0.03186	0.031916	tanh	true	0.00061091	[192 62 5]
14	Accept	0.044707	848.71	0.03186	0.03201	tanh	true	3.65E-07	300
15	Accept	0.042612	526.53	0.03186	0.032006	relu	true	4.14E-10	240
16	Accept	0.15665	37.948	0.03186	0.031996	relu	true	4.51E-10	1
17	Accept	0.042414	597.43	0.03186	0.03198	relu	true	7.93E-08	258
18	Accept	0.49992	9.3932	0.03186	0.032061	relu	true	0.13161	[282 2]
19	Accept	0.10977	880.93	0.03186	0.032065	sigmoid	true	0.0010603	[271 61 30]
20	Accept	0.49992	0.71501	0.03186	0.032141	sigmoid	true	3.0824	[1 17]
21	Accept	0.041742	938.96	0.03186	0.032138	sigmoid	true	9.12E-07	[299 61 3]
22	Accept	0.046881	751.16	0.03186	0.032143	sigmoid	true	6.06E-10	[296 7 3]
23	Accept	0.45415	62.968	0.03186	0.032188	relu	false	5.36E-10	[1 21 80]
24	Accept	0.49992	19.686	0.03186	0.032194	relu	false	2.7933	[279 11]
25	Accept	0.12234	1563.8	0.03186	0.032186	sigmoid	false	7.27E-10	[282 112 223]
26	Accept	0.1157	596.51	0.03186	0.032159	sigmoid	false	3.81E-05	243
27	Accept	0.43762	8.9281	0.03186	0.032226	sigmoid	false	1.77E-07	1
28	Accept	0.49992	11.07	0.03186	0.032243	sigmoid	false	3.0743	[298 86]
29	Accept	0.039924	65.818	0.03186	0.032274	relu	true	3.35E-08	22
30	Accept	0.036287	821.7	0.03186	0.032335	tanh	true	2.36E-05	[44 15 273]
31	Accept	0.1091	783.6	0.03186	0.032193	tanh	false	4.25E-10	281
32	Accept	0.1104	608.51	0.03186	0.032279	tanh	false	1.22E-05	220
33	Accept	0.31117	282.61	0.03186	0.032262	tanh	false	5.89E-08	[1 166]

34	Accept	0.49992	15.293	0.03186	0.032277	tanh	false	1.1	[289 44]
35	Accept	0.040003	706.96	0.03186	0.032249	sigmoid	true	1.59E-08	[288 1 16]
36	Accept	0.15132	475.63	0.03186	0.032228	none	true	5.13E-10	[287 36]
37	Accept	0.15187	1126.6	0.03186	0.032219	none	true	0.00014384	[290 192 112]
38	Accept	0.15124	30.342	0.03186	0.032201	none	true	2.83E-07	1
39	Accept	0.49992	0.50495	0.03186	0.032226	none	true	3.6729	1
40	Accept	0.18254	544.97	0.03186	0.032209	none	false	3.96E-10	[280 10 2]
41	Accept	0.1863	1303.4	0.03186	0.032247	none	false	0.00041739	[271 253 7]
42	Accept	0.22191	63.263	0.03186	0.032242	none	false	3.53E-07	[1 9 8]
43	Accept	0.49992	1.3065	0.03186	0.032214	none	false	1.2755	[1 9]
44	Accept	0.11151	844.38	0.03186	0.032284	tanh	false	7.12E-08	[299 8]
45	Accept	0.041703	619.04	0.03186	0.032317	relu	true	6.17E-09	289
46	Accept	0.15128	463.99	0.03186	0.032325	none	true	3.40E-07	[296 8]
47	Accept	0.041189	684.09	0.03186	0.03238	tanh	true	3.98E-10	[294 29]
48	Accept	0.15124	25.422	0.03186	0.032187	none	true	5.12E-10	1
49	Accept	0.040161	350.68	0.03186	0.032668	sigmoid	true	4.42E-10	[10 141]
50	Accept	0.11479	703.59	0.03186	0.032298	sigmoid	false	1.95E-07	[286 2]
51	Accept	0.032493	817.92	0.03186	0.031887	tanh	true	0.00024342	[82 221 9]
52	Accept	0.22018	31.46	0.03186	0.032042	relu	true	2.96E-07	[1 3]
53	Accept	0.042414	422.16	0.03186	0.032041	relu	true	1.39E-09	[46 45 119]
54	Accept	0.041387	172.55	0.03186	0.032	relu	true	1.33E-06	[51 24]
55	Accept	0.046486	155.87	0.03186	0.031992	sigmoid	true	2.85E-09	[49 8]
56	Accept	0.18555	562.08	0.03186	0.031986	none	false	2.67E-07	290
57	Accept	0.045142	235.76	0.03186	0.027613	relu	true	5.88E-08	[65 37]
58	Accept	0.14867	28.821	0.03186	0.027574	sigmoid	true	4.34E-10	1
59	Accept	0.034746	91.867	0.03186	0.027409	sigmoid	true	5.76E-06	32
60	Accept	0.040438	275.04	0.03186	0.027414	sigmoid	true	4.17E-07	[53 2 62]
61	Accept	0.041426	122.88	0.03186	0.027567	sigmoid	true	3.96E-10	[40 5]
62	Accept	0.034983	338.1	0.03186	0.027798	sigmoid	true	1.15E-05	135
63	Accept	0.49992	8.1222	0.03186	0.032002	relu	false	1.04E-07	[293 2]
64	Accept	0.19701	151.94	0.03186	0.032142	relu	false	0.00037342	[1 22 31]
65	Accept	0.041031	553.2	0.03186	0.03214	relu	true	6.33E-06	245
66	Accept	0.035932	451.76	0.03186	0.032157	tanh	true	4.79E-07	[26 1 165]
67	Accept	0.034667	620.15	0.03186	0.030084	sigmoid	true	1.77E-05	[37 241]
68	Accept	0.044272	235.01	0.03186	0.028956	tanh	true	2.10E-06	77
69	Accept	0.068345	47.85	0.03186	0.032115	tanh	true	4.36E-08	4
70	Accept	0.040754	760.9	0.03186	0.029003	tanh	true	7.88E-09	[208 1 68]
71	Accept	0.039292	144.71	0.03186	0.032041	relu	true	1.69E-05	[43 13]
72	Accept	0.043917	160.83	0.03186	0.031177	relu	true	3.94E-09	58
73	Accept	0.2182	50.829	0.03186	0.030849	none	false	5.01E-10	[1 5]
74	Best	0.031149	370.29	0.031149	0.030688	tanh	true	0.00049278	[77 32 15]
75	Accept	0.040122	751.86	0.031149	0.030683	sigmoid	true	4.80E-06	[80 201]
76	Accept	0.073365	45.293	0.031149	0.03074	sigmoid	true	1.87E-05	3
77	Best	0.030912	797.6	0.030912	0.030663	sigmoid	true	2.24E-05	[298 12]
78	Accept	0.03356	145.23	0.030912	0.03067	tanh	true	0.00013468	[27 15]
79	Accept	0.043679	329.82	0.030912	0.030672	tanh	true	4.14E-10	[99 4]
80	Accept	0.042849	726.4	0.030912	0.030668	relu	true	1.06E-06	[289 16 1]
81	Accept	0.040398	73.69	0.030912	0.030643	tanh	true	3.80E-06	[14 1]
82	Accept	0.13673	407.53	0.030912	0.030641	relu	false	0.00072907	[56 92]
83	Accept	0.099731	770.62	0.030912	0.025727	tanh	true	0.0021598	291
84	Accept	0.03522	302.43	0.030912	0.027433	tanh	true	0.00016108	94
85	Accept	0.035615	333.63	0.030912	0.029763	tanh	true	0.00019718	[101 3 2]
86	Accept	0.033402	395.78	0.030912	0.030755	tanh	true	0.00028335	[51 23 63]
87	Accept	0.033125	877.02	0.030912	0.030941	sigmoid	true	4.07E-05	[136 28 203]
88	Accept	0.032335	1071.8	0.030912	0.031444	sigmoid	true	3.29E-05	[297 81]
89	Accept	0.044589	409.74	0.030912	0.0311	relu	true	5.26E-06	[81 84 3]
90	Accept	0.042691	459.46	0.030912	0.030021	sigmoid	true	2.23E-06	[12 2 179]
91	Accept	0.033837	1191.2	0.030912	0.031567	tanh	true	0.00029088	[134 260]
92	Accept	0.036367	506.71	0.030912	0.031621	tanh	true	2.98E-09	[17 5 167]
93	Accept	0.038303	73.486	0.030912	0.031678	relu	true	4.18E-10	22
94	Accept	0.035932	726.91	0.030912	0.031636	sigmoid	true	2.23E-05	[267 2 1]
95	Accept	0.045735	774.76	0.030912	0.031575	sigmoid	true	1.21E-07	291
96	Accept	0.035971	877.95	0.030912	0.031893	tanh	true	0.00011133	[288 9 5]
97	Accept	0.25694	56.357	0.030912	0.031891	tanh	false	0.00081644	[1 1 7]
98	Accept	0.03103	198.4	0.030912	0.031282	tanh	true	0.00031143	[49 3 8]
99	Accept	0.038303	430.8	0.030912	0.03128	tanh	true	7.69E-08	[23 48 71]
100	Accept	0.032137	725.73	0.030912	0.031274	tanh	true	0.00043991	[105 5 154]

TPEF-SHG model

Iter	Eval result	Objective	Objective runtime	BestSoFar (observed)	BestSoFar (estim)	Activations	Standardize	Lambda	LayerSizes
1	Best	0.05032	844.76	0.05032	0.05032	relu	false	4.01E-05	[291 35]
2	Best	0.036485	61.982	0.036485	0.037035	sigmoid	true	2.83E-08	[2 6 8]
3	Best	0.014784	438.53	0.014784	0.017936	tanh	true	3.41E-09	[59 264]
4	Accept	0.033599	32.436	0.014784	0.016758	relu	true	2.28E-05	2

5	Best	0.014388	377.74	0.014388	0.014451	tanh	true	5.09E-08	[64 238]
6	Best	0.014033	489.54	0.014033	0.014164	tanh	true	1.67E-09	[295 3]
7	Accept	0.49992	2.744	0.014033	0.014405	sigmoid	true	0.20609	[95 1]
8	Accept	0.41655	140.96	0.014033	0.01431	sigmoid	true	0.0031544	[229 1 48]
9	Best	0.012412	112.45	0.012412	0.012422	relu	true	5.14E-05	[45 1]
10	Accept	0.046051	48.161	0.012412	0.012422	tanh	true	1.28E-07	[1 1 7]
11	Accept	0.49992	20.049	0.012412	0.012433	relu	true	0.27312	[300 292 16]
12	Accept	0.012649	104.51	0.012412	0.012611	tanh	true	1.76E-05	[23 7]
13	Accept	0.014547	664.1	0.012412	0.012646	sigmoid	true	2.54E-07	[70 29 198]
14	Accept	0.017551	678.28	0.012412	0.012633	sigmoid	true	4.58E-10	[7 288 15]
15	Accept	0.49992	0.35019	0.012412	0.012662	relu	false	4.06E-10	1
16	Accept	0.49992	7.8251	0.012412	0.012648	relu	false	2.1746	[3 299 4]
17	Accept	0.019251	687.54	0.012412	0.012634	tanh	true	0.00097076	[5 36 254]
18	Accept	0.015337	654.42	0.012412	0.012595	relu	true	7.09E-08	[94 3 143]
19	Accept	0.014428	104.38	0.012412	0.012619	tanh	true	3.97E-10	[45 34]
20	Accept	0.49992	8.9912	0.012412	0.012581	tanh	true	3.923	[262 27 187]
21	Accept	0.014112	142.25	0.012412	0.012645	relu	true	3.96E-06	[40 15 2]
22	Accept	0.014468	250.07	0.012412	0.012635	relu	true	4.03E-10	[208 93 19]
23	Accept	0.054352	942.86	0.012412	0.012642	sigmoid	false	3.97E-10	[110 3 294]
24	Accept	0.054313	399.27	0.012412	0.012641	sigmoid	false	2.22E-06	[154 2]
25	Accept	0.1759	2.8069	0.012412	0.012646	sigmoid	false	0.066103	1
26	Accept	0.039529	839.54	0.012412	0.01264	tanh	false	5.25E-08	[284 7]
27	Accept	0.045616	285.64	0.012412	0.012608	tanh	false	0.00014471	[85 18]
28	Accept	0.33228	39.565	0.012412	0.012633	tanh	false	2.3994	165
29	Accept	0.085501	48.062	0.012412	0.012623	tanh	false	2.45E-06	[1 5]
30	Accept	0.042177	807.71	0.012412	0.012601	tanh	false	3.99E-10	[257 16 16]
31	Accept	0.09392	377.74	0.012412	0.012618	none	false	2.01E-08	[2 4 182]
32	Accept	0.088545	558.16	0.012412	0.012613	none	false	0.00054929	294
33	Accept	0.49992	2.4369	0.012412	0.012598	none	false	3.9392	[2 36]
34	Accept	0.088782	54.103	0.012412	0.012599	none	false	4.60E-06	12
35	Accept	0.012412	274.72	0.012412	0.012291	tanh	true	0.00019364	94
36	Accept	0.047593	912.28	0.012412	0.012295	none	true	3.99E-10	[189 290]
37	Accept	0.047395	125.48	0.012412	0.012298	none	true	1.25E-06	66
38	Accept	0.091153	1.6328	0.012412	0.012301	none	true	0.018638	1
39	Accept	0.41636	7.4716	0.012412	0.012468	sigmoid	false	2.97E-08	[1 1]
40	Accept	0.014626	282.77	0.012412	0.012467	sigmoid	true	3.76E-09	[293 17]
41	Accept	0.049055	33.372	0.012412	0.01246	none	true	8.12E-09	[1 3]
42	Accept	0.059768	100.25	0.012412	0.012451	relu	true	4.33E-10	[1 27]
43	Accept	0.0453	795.71	0.012412	0.012366	sigmoid	false	0.00047437	[296 4 17]
44	Accept	0.040794	696.52	0.012412	0.012472	sigmoid	false	2.53E-05	282
45	Accept	0.088386	536.52	0.012412	0.012466	none	false	1.05E-08	280
46	Accept	0.49992	5.9806	0.012412	0.012476	none	true	3.927	[251 134 2]
47	Accept	0.047435	27.341	0.012412	0.012479	none	true	2.28E-05	1
48	Accept	0.49992	8.5883	0.012412	0.012364	sigmoid	false	3.9088	[297 62]
49	Accept	0.048304	549.41	0.012412	0.012362	none	true	0.00070263	[284 67]
50	Accept	0.014072	280.3	0.012412	0.012358	relu	true	4.41E-09	[283 61 17]
51	Accept	0.25239	45.811	0.012412	0.012442	tanh	false	4.47E-09	[1 17]
52	Accept	0.041821	864.47	0.012412	0.012449	tanh	false	4.07E-06	300
53	Accept	0.047039	36.958	0.012412	0.012407	tanh	true	0.00010692	1
54	Best	0.01174	802.51	0.01174	0.011744	tanh	true	2.96E-06	[283 5]
55	Accept	0.086252	61.654	0.01174	0.011743	tanh	false	0.00062353	[1 10]
56	Accept	0.046012	45.752	0.01174	0.01174	tanh	true	4.03E-10	[1 7]
57	Accept	0.014942	848.92	0.01174	0.01174	sigmoid	true	5.41E-08	[298 209 4]
58	Accept	0.045537	1340.7	0.01174	0.011738	sigmoid	false	5.38E-09	[298 191]
59	Best	0.010475	1285.3	0.010475	0.010451	relu	true	1.27E-05	[280 210]
60	Accept	0.015535	819.51	0.010475	0.010446	sigmoid	true	4.01E-10	[276 293]
61	Accept	0.011424	772.08	0.010475	0.010438	tanh	true	7.15E-05	[45 12 257]
62	Accept	0.047988	41.212	0.010475	0.010437	none	true	4.57E-10	[1 11]
63	Accept	0.17061	63.457	0.010475	0.010435	relu	false	3.15E-05	[1 21]
64	Accept	0.05202	14	0.010475	0.01044	none	true	0.00059279	1
65	Accept	0.047632	543.9	0.010475	0.010438	relu	true	6.93E-08	[1 136 71]
66	Accept	0.12084	318.51	0.010475	0.010441	none	false	4.22E-10	[1 162]
67	Accept	0.015298	97.113	0.010475	0.010424	sigmoid	true	2.32E-08	[44 6]
68	Accept	0.044984	713.79	0.010475	0.010446	sigmoid	false	4.09E-10	296
69	Accept	0.012807	127.56	0.010475	0.010529	relu	true	1.13E-05	50
70	Accept	0.01257	629.75	0.010475	0.01051	relu	true	7.78E-07	[269 7 1]
71	Accept	0.013716	469.34	0.010475	0.010537	sigmoid	true	5.92E-09	[118 24 269]
72	Accept	0.047316	1087.6	0.010475	0.010537	none	true	3.41E-05	[290 237]
73	Accept	0.014468	172.48	0.010475	0.010539	tanh	true	1.09E-06	[50 4]
74	Accept	0.088624	996.84	0.010475	0.010535	none	false	3.97E-06	[289 157]
75	Accept	0.052218	1326.1	0.010475	0.010538	relu	false	1.90E-06	[298 115 79]
76	Accept	0.016049	228.32	0.010475	0.010531	relu	true	2.01E-09	[38 31 143]
77	Accept	0.012293	521.49	0.010475	0.010532	tanh	true	1.86E-05	185
78	Accept	0.01257	787.51	0.010475	0.010532	tanh	true	1.28E-07	[283 45]
79	Accept	0.047553	657.12	0.010475	0.010533	none	true	1.87E-08	[296 93]
80	Accept	0.010791	351.09	0.010475	0.010532	tanh	true	0.00032024	[22 112 6]
81	Accept	0.04613	33.777	0.010475	0.01053	sigmoid	true	1.77E-06	[1 2]
82	Accept	0.013756	649.57	0.010475	0.01053	tanh	true	3.98E-10	[288 1 1]

83	Accept	0.016444	348.01	0.010475	0.010526	sigmoid	true	4.45E-10	[40 2 152]
84	Accept	0.010989	871.62	0.010475	0.010376	relu	true	4.16E-05	[241 124 2]
85	Accept	0.013203	373.54	0.010475	0.010334	tanh	true	2.09E-08	[266 15 3]
86	Accept	0.01257	897.58	0.010475	0.010952	relu	true	1.07E-05	[145 2 259]
87	Accept	0.01257	368.25	0.010475	0.01094	tanh	true	6.43E-06	[78 51]
88	Accept	0.045972	47.175	0.010475	0.010686	sigmoid	true	4.54E-10	[1 9]
89	Accept	0.011345	519.04	0.010475	0.010986	relu	true	2.61E-05	[72 156]
90	Accept	0.016839	157.91	0.010475	0.010985	sigmoid	true	1.84E-07	[18 46]
91	Accept	0.012452	563.39	0.010475	0.010968	relu	true	7.76E-08	297
92	Accept	0.047948	77.735	0.010475	0.010962	none	true	0.0001627	[21 14 3]
93	Accept	0.016997	95.184	0.010475	0.010964	sigmoid	true	2.05E-09	[21 10]
94	Accept	0.010831	640.85	0.010475	0.010536	relu	true	2.16E-05	288
95	Accept	0.013163	105.73	0.010475	0.010542	tanh	true	0.00019726	31
96	Accept	0.012728	141.73	0.010475	0.010547	tanh	true	0.00081048	[39 7]
97	Accept	0.012175	1082.5	0.010475	0.010545	tanh	true	7.34E-07	[298 84]
98	Accept	0.30429	124.66	0.010475	0.010409	relu	true	2.99E-05	[129 1 1]
99	Accept	0.013321	1111.1	0.010475	0.01042	relu	true	4.67E-06	[93 91 292]
100	Accept	0.042375	1061.5	0.010475	0.010421	tanh	false	1.33E-06	[276 1 129]

CARS-TPEF-SHG model

Iter	Eval result	Objective	Objective runtime	BestSoFar (observed)	BestSoFar (estim)	Activations	Standardize	Lambda	LayerSizes
1	Best	0.03862	926.61	0.03862	0.03862	relu	false	4.01E-05	[291 35]
2	Best	0.021701	72.866	0.021701	0.022374	sigmoid	true	2.83E-08	[2 6 8]
3	Best	0.010357	155.88	0.010357	0.010369	tanh	true	3.41E-09	[59 264]
4	Accept	0.018776	44.75	0.010357	0.011216	relu	true	2.28E-05	2
5	Accept	0.011266	111.71	0.010357	0.010376	tanh	true	5.22E-10	[65 180]
6	Accept	0.49992	1.8346	0.010357	0.010397	tanh	true	2.7319	[22 50]
7	Accept	0.49992	0.77599	0.010357	0.010371	relu	true	3.0231	3
8	Best	0.0096055	115.13	0.0096055	0.0096067	relu	true	9.22E-08	[24 3]
9	Best	0.0072338	1333.3	0.0072338	0.0072456	sigmoid	true	0.00010193	[215 129 204]
10	Accept	0.49992	1.6393	0.0072338	0.0072536	sigmoid	true	3.2443	[5 51]
11	Accept	0.015614	293.07	0.0072338	0.0072809	sigmoid	true	6.18E-08	[3 4 116]
12	Accept	0.3252	199.69	0.0072338	0.0072759	relu	false	3.27E-09	[155 2]
13	Accept	0.17946	341.76	0.0072338	0.0072726	relu	false	0.054029	[160 5]
14	Accept	0.017867	46.442	0.0072338	0.0073147	sigmoid	true	5.83E-06	2
15	Accept	0.018302	670.65	0.0072338	0.0073189	relu	true	1.48E-06	[2 294]
16	Accept	0.01846	47.398	0.0072338	0.0073264	relu	true	4.03E-10	2
17	Accept	0.058305	361.82	0.0072338	0.0073194	sigmoid	false	9.13E-10	[26 64 39]
18	Accept	0.065064	154.18	0.0072338	0.0073163	sigmoid	false	1.43E-05	[8 4 34]
19	Accept	0.49992	9.2365	0.0072338	0.0073267	sigmoid	false	2.6149	166
20	Accept	0.053601	81.797	0.0072338	0.0073335	sigmoid	false	1.36E-07	16
21	Accept	0.04107	575.12	0.0072338	0.0073452	tanh	false	4.09E-10	[81 9 110]
22	Accept	0.05119	123.51	0.0072338	0.0073255	tanh	false	1.88E-06	33
23	Accept	0.49992	10.506	0.0072338	0.0073355	tanh	false	0.36168	[277 8 18]
24	Accept	0.41268	11.488	0.0072338	0.0072582	tanh	false	2.85E-08	[1 2]
25	Best	0.0071152	370.63	0.0071152	0.0071156	sigmoid	true	5.83E-07	[147 51]
26	Accept	0.032414	1226.9	0.0071152	0.0071154	tanh	false	5.46E-08	[295 97 1]
27	Accept	0.012452	166.6	0.0071152	0.0071143	tanh	true	4.45E-10	[3 44]
28	Accept	0.0094869	59.33	0.0071152	0.0071128	relu	true	4.14E-10	[119 6]
29	Accept	0.15313	148.69	0.0071152	0.0071131	sigmoid	false	1.33E-08	[1 2 54]
30	Best	0.0069571	117.19	0.0069571	0.0071357	sigmoid	true	8.22E-06	37
31	Accept	0.078425	3.6308	0.0069571	0.0071339	sigmoid	true	0.0078217	3
32	Accept	0.0072338	78.637	0.0069571	0.007136	relu	true	1.47E-07	65
33	Accept	0.024547	50.665	0.0069571	0.0071258	sigmoid	true	9.96E-10	[2 2]
34	Accept	0.032809	2035.9	0.0069571	0.0069232	tanh	false	2.77E-06	[274 299]
35	Accept	0.011068	200.39	0.0069571	0.0071234	sigmoid	true	5.13E-10	[292 7 4]
36	Accept	0.050083	1362.4	0.0069571	0.0069376	tanh	false	4.11E-10	[19 285 125]
37	Accept	0.02099	27.474	0.0069571	0.0069428	sigmoid	true	0.00022854	4
38	Accept	0.010673	160.91	0.0069571	0.0071305	sigmoid	true	5.04E-09	[41 238]
39	Accept	0.041031	1121.3	0.0069571	0.0071301	tanh	false	0.0006377	[86 295]
40	Accept	0.49992	88.268	0.0069571	0.0071192	sigmoid	false	0.029998	[33 236 250]
41	Accept	0.06542	65.12	0.0069571	0.0069489	tanh	false	4.68E-05	[2 2]
42	Accept	0.032769	782.85	0.0069571	0.00694	tanh	false	5.18E-05	[264 1 14]
43	Best	0.0062851	655.11	0.0062851	0.0062971	relu	true	1.70E-05	[284 5]
44	Accept	0.15282	72.232	0.0062851	0.0062969	relu	false	8.95E-05	[1 22]
45	Accept	0.032414	755.68	0.0062851	0.0062964	sigmoid	false	1.34E-06	294
46	Accept	0.033916	459.41	0.0062851	0.0062964	tanh	true	1.52E-07	[1 193]
47	Accept	0.0071942	614.28	0.0062851	0.006296	tanh	true	1.16E-06	[279 8]
48	Accept	0.0087359	147.1	0.0062851	0.0062966	tanh	true	8.18E-08	[289 14 12]
49	Accept	0.035299	159.03	0.0062851	0.006296	none	true	7.16E-09	[54 36]
50	Accept	0.035102	202.4	0.0062851	0.0062959	none	true	2.49E-06	[1 119]
51	Accept	0.035102	1209.5	0.0062851	0.006296	none	true	1.68E-06	[293 183 124]

52	Accept	0.056882	7.8791	0.0062851	0.006296	none	true	0.0042598	15
53	Accept	0.49992	3.6885	0.0062851	0.006296	none	true	3.8307	[295 1]
54	Accept	0.036485	29.82	0.0062851	0.0062962	none	true	0.00021203	1
55	Accept	0.035615	263.3	0.0062851	0.0062965	none	true	0.00013573	[130 25]
56	Accept	0.035339	38.727	0.0062851	0.0062965	none	true	4.17E-10	1
57	Accept	0.03526	37.858	0.0062851	0.0062967	none	true	3.53E-08	1
58	Accept	0.067239	494.59	0.0062851	0.0062963	none	false	5.02E-10	[81 107 54]
59	Accept	0.071784	565.1	0.0062851	0.0062962	none	false	8.80E-07	[1 298]
60	Accept	0.067199	581.83	0.0062851	0.006296	none	false	0.00066775	288
61	Accept	0.065895	1562.2	0.0062851	0.0062959	none	false	3.11E-07	[292 293]
62	Accept	0.49992	6.1164	0.0062851	0.0062967	none	false	1.3943	[1 7 112]
63	Accept	0.13495	61.744	0.0062851	0.0062963	none	false	4.70E-10	[1 8]
64	Accept	0.0083801	146.22	0.0062851	0.0062969	relu	true	6.56E-09	[257 98 77]
65	Accept	0.03103	764.59	0.0062851	0.0062966	sigmoid	false	8.58E-09	298
66	Accept	0.010357	134.16	0.0062851	0.0062969	sigmoid	true	2.35E-08	287
67	Accept	0.0073128	93.586	0.0062851	0.0063181	relu	true	5.52E-06	32
68	Accept	0.0083406	99.727	0.0062851	0.0063179	tanh	true	3.01E-07	39
69	Accept	0.066408	886.85	0.0062851	0.0063182	none	false	2.43E-05	[246 150]
70	Accept	0.010277	39.114	0.0062851	0.0063182	relu	true	2.57E-09	[20 8]
71	Best	0.0061665	859.31	0.0061665	0.0061527	relu	true	1.27E-06	[283 6 186]
72	Accept	0.037592	779.78	0.0061665	0.0061523	sigmoid	false	3.97E-10	[299 1]
73	Accept	0.034232	42.357	0.0061665	0.0061528	tanh	true	1.77E-05	1
74	Accept	0.035299	475.13	0.0061665	0.0061533	none	true	4.18E-10	[283 1 13]
75	Accept	0.006206	736.19	0.0061665	0.0061519	tanh	true	3.84E-05	264
76	Accept	0.0071942	133.4	0.0061665	0.0061595	tanh	true	8.89E-06	[36 2]
77	Accept	0.011187	112.08	0.0061665	0.0061553	tanh	true	2.35E-09	277
78	Accept	0.0069966	723.86	0.0061665	0.0061574	sigmoid	true	1.23E-05	[294 3]
79	Accept	0.032928	695.38	0.0061665	0.0061504	sigmoid	true	0.0010156	[286 4]
80	Accept	0.036683	136.38	0.0061665	0.0061642	relu	true	1.31E-08	[1 37]
81	Accept	0.0070361	828.05	0.0061665	0.0061592	tanh	true	1.03E-05	[300 9]
82	Accept	0.052376	642.59	0.0061665	0.0061754	tanh	false	0.00010132	[24 192 2]
83	Accept	0.033323	782.42	0.0061665	0.0061787	tanh	false	7.34E-07	[287 1]
84	Accept	0.065934	160.81	0.0061665	0.006179	none	false	0.00013704	[1 73]
85	Accept	0.06961	624.9	0.0061665	0.0061785	none	false	7.41E-09	[287 4]
86	Accept	0.0066408	517.17	0.0061665	0.0061975	relu	true	0.00036913	[232 2]
87	Accept	0.03356	1963.7	0.0061665	0.0061938	tanh	false	4.31E-10	[287 245 1]
88	Accept	0.0070757	225.83	0.0061665	0.0061868	relu	true	0.00010685	[51 37]
89	Accept	0.038896	1712	0.0061665	0.0061875	relu	false	2.26E-06	[274 211]
90	Accept	0.0064827	687.52	0.0061665	0.0061717	relu	true	0.00010814	[270 4 9]
91	Accept	0.12811	51.587	0.0061665	0.0061335	relu	true	0.00057252	[1 3 3]
92	Accept	0.011621	668.15	0.0061665	0.0061272	sigmoid	true	6.46E-07	[9 283 1]
93	Accept	0.033916	46.894	0.0061665	0.0061261	tanh	true	3.29E-09	1
94	Accept	0.49992	2.6869	0.0061665	0.006095	relu	false	3.58	[1 54 61]
95	Accept	0.0080243	120.52	0.0061665	0.0060947	relu	true	3.80E-08	295
96	Accept	0.0084987	74.637	0.0061665	0.0060875	sigmoid	true	1.04E-07	52
97	Accept	0.0074709	125.76	0.0061665	0.0060894	tanh	true	0.00020855	[14 14 6]
98	Accept	0.0073524	824.5	0.0061665	0.0060843	tanh	true	0.00043832	[288 2]
99	Accept	0.0064827	1018.2	0.0061665	0.0060816	tanh	true	0.00013867	[107 235]
100	Accept	0.036841	73.34	0.0061665	0.0060748	tanh	true	0.0011986	[1 13]

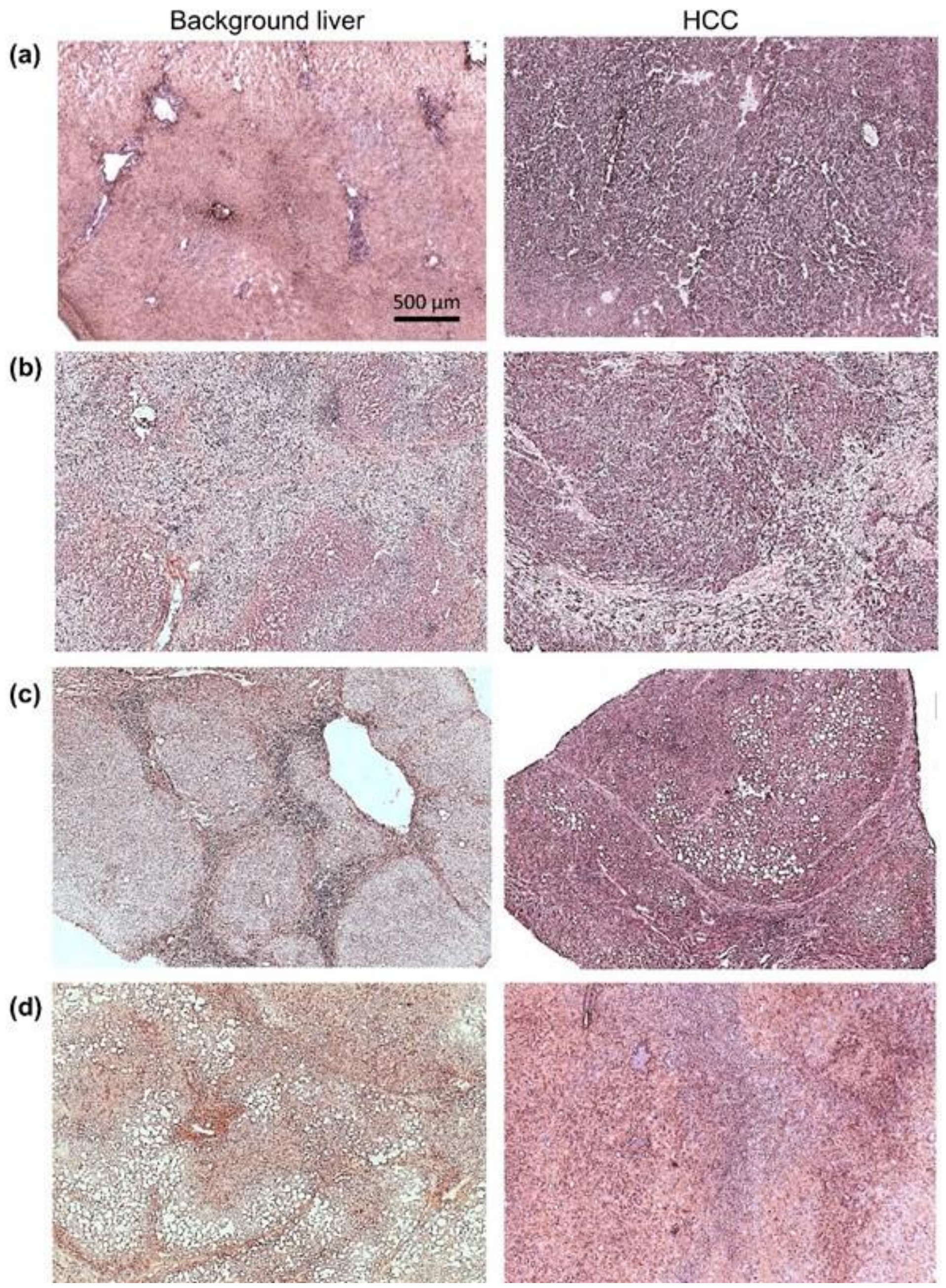

Figure S2: HE staining images of the samples shown in Figure 1.

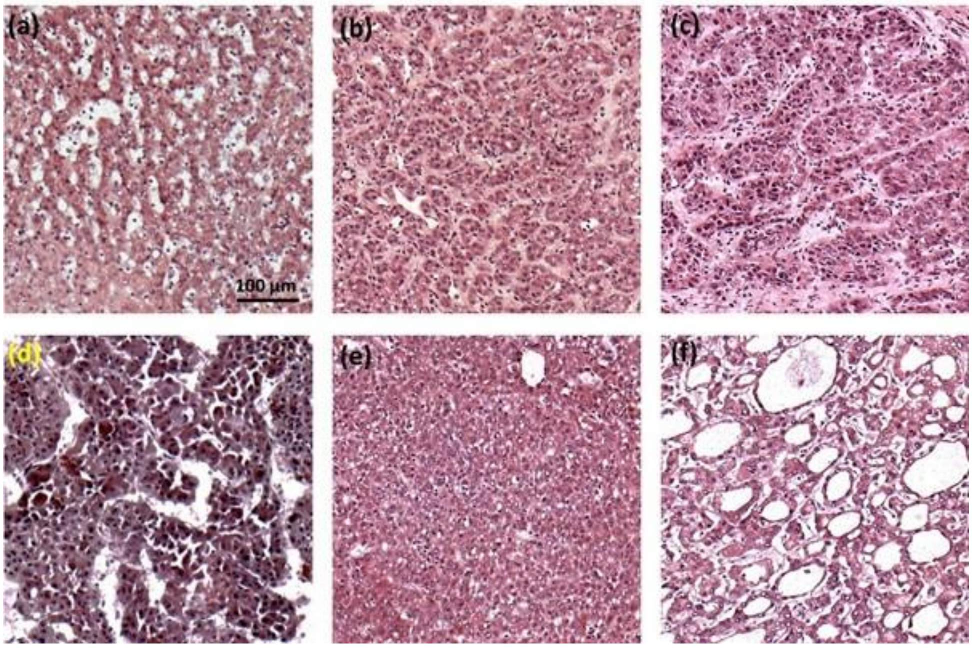

Figure S3: He staining images of samples in Figure 2, showing growth patterns of ordinary HCC. (a) normal liver parenchyma as reference; (b) – (c): trabecular; (d) macrotrabecular; (e) solid; (f) acinar.

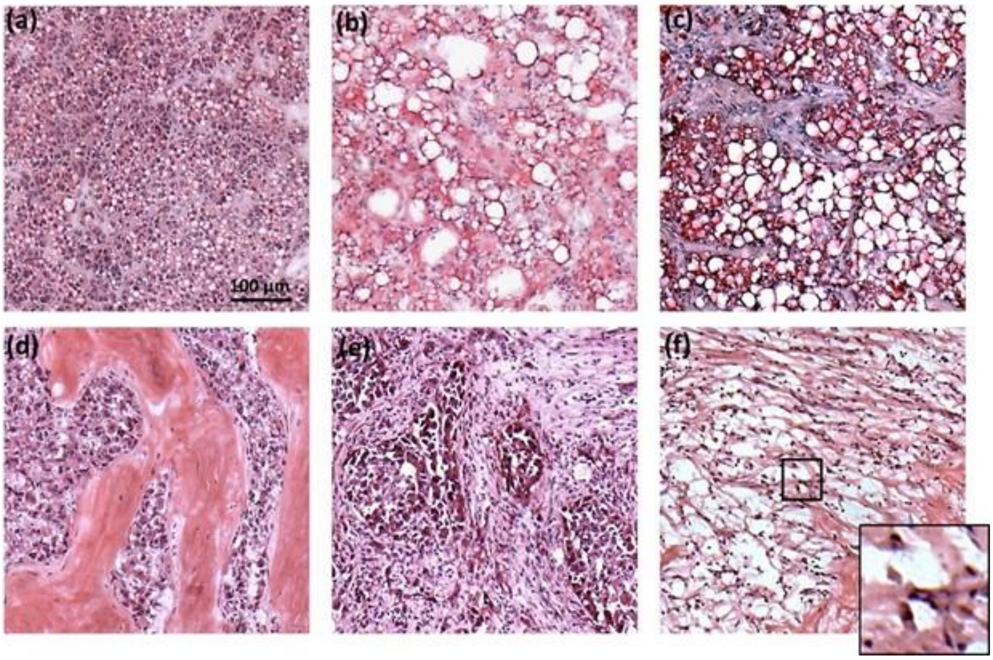

Figure S4: HE staining images of samples in Figure 4, with fatty changes and with increased stromal reactions.

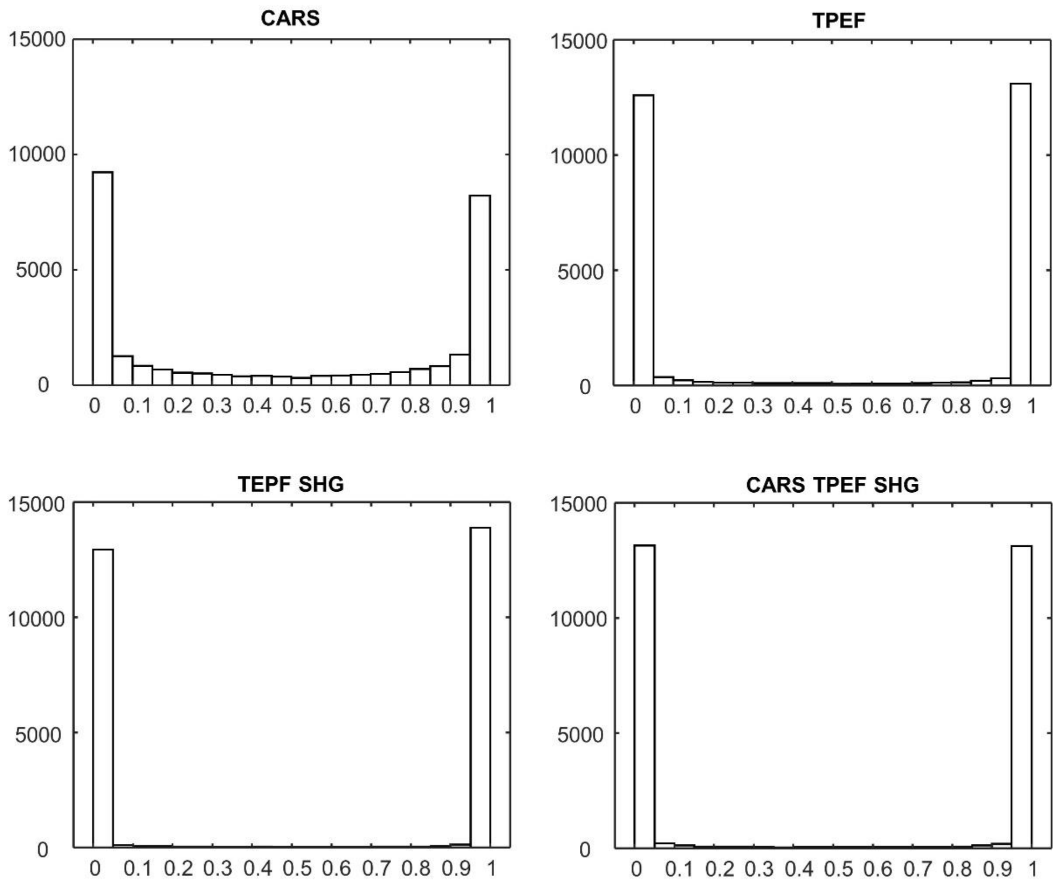

Figure S5: posterior probabilities of selected classification models applied on the test set A (normal tissue: posterior probability = 0; tumor: posterior probability = 1).

Table S5: Fraction of correctly classified FoV images vs. tumor differentiation grade (test set A).

Tumor grade	Classification models						
	CARS	TPEF	SHG	CARS-TPEF	CARS-SHG	TPEF-SHG	CARS-TPEF-SHG
G1	0.654	0.757	0.897	0.757	0.870	0.939	0.827
G2	0.683	0.945	0.925	0.929	0.861	0.964	0.950
G3	0.812	0.995	0.879	0.974	0.874	0.989	0.959

G1: N. of FoV images: 2058
G2: N. of FoV images: 10189
G3: N. of FoV images: 2657
